# Dual prophylactic and therapeutic potential of iPSC-based vaccines and neoantigen discovery in colorectal cancer

**DOI:** 10.7150/thno.111400

**Published:** 2025-04-28

**Authors:** Si-Han Jwo, Shang-Kok Ng, Chin-Tzu Li, Shao-Peng Chen, Li-Yu Chen, Pin-Jung Liu, Huai-Jie Wang, Jr-Shiuan Lin, Chun-Jung Ko, Cheng-Fan Lee, Chun-Hao Wang, Xiaoming Ouyang, Lin Wang, Tzu-Tang Wei

**Affiliations:** 1 Department and Graduate Institute of Pharmacology, College of Medicine, National Taiwan University, Taipei 10051, Taiwan.; 2 School of Pharmacy, College of Pharmacy, Taipei Medical University, Taipei 11031, Taiwan.; 3 Graduate Institute of Immunology, College of Medicine, National Taiwan University, Taipei 10051, Taiwan.; 4 Department of Biochemistry and Molecular Cell Biology, School of Medicine, College of Medicine, Taipei Medical University, Taipei 11031, Taiwan.; 5 Department of Internal Medicine, National Taiwan University Hospital, Taipei 100225, Taiwan.; 6 Stanford Cardiovascular Institute, Stanford University, Stanford, CA 94305, USA.; 7 Chemical Biology and Molecular Biophysics, Taiwan International Graduate Program in Chemical Biology and Molecular Biophysics (TIGP-CBMB), Academia Sinica, Taipei 11529, Taiwan.

**Keywords:** induced pluripotent stem cells (iPSC), colorectal cancer (CRC), cancer vaccine, LC-MS, neoantigens

## Abstract

**Rationale:** Induced pluripotent stem cells (iPSCs) share transcriptomic similarities with cancer cells and express tumor-specific and tumor-associated antigens, highlighting their potential as cancer vaccines. Our previous study demonstrated that an iPSC-based vaccine effectively prevented tumor growth in various mouse models, including melanoma, breast, lung, and pancreatic cancers. However, the underlying mechanisms and the therapeutic efficacy of the iPSC-based vaccine remain unclear. Colorectal cancer (CRC), the third most common cancer with a rising incidence worldwide, presents an urgent need for novel strategies to prevent and treat CRC.

**Methods:** Allograft mouse models were established to evaluate the antitumor effects of the iPSC-based vaccine. CpG oligonucleotide (ODN) 1826 served as a vaccine adjuvant. Bulk RNA-Sequencing (RNA-Seq) and the Microenvironment Cell Population counter (MCP-Counter) algorithm were performed to analyze transcriptomic changes. Liquid chromatography-mass spectrometry (LC-MS) combined with in silico strategies was employed to identify potential antigen proteins. Chinese Hamster Ovary (CHO-K1) models were utilized to express candidate neoantigen proteins. Mouse bone marrow-derived dendritic cells (BMDCs) were used to investigate T cell priming in response to iPSC-associated proteins. Immune cell profiles were characterized by flow cytometry.

**Results:** The combination of CpG and iPSC vaccination demonstrated both prophylactic and therapeutic efficacy in reducing tumor growth in CRC mouse models. Vaccination significantly increased CD8^+^ T cell infiltration within tumor regions, while T cell depletion abrogated the antitumor effects, underscoring the critical role of T cells in mediating these responses. Proteomic analysis identified two iPSC-associated proteins, heterogeneous nuclear ribonucleoprotein U (HNRNPU) and nucleolin (NCL), as key drivers of the observed immune responses. Vaccination with HNRNPU or NCL, in combination with CpG, enhanced dendritic cell activation, induced antigen-specific CD8^+^ T cell cytotoxicity, and promoted the formation of central memory CD8^+^ T cells, collectively leading to significant CRC tumor shrinkage.

**Conclusions:** Our findings reveal potential mechanisms underlying the efficacy of iPSC-based vaccines in cancer immunotherapy. Additionally, HNRNPU and NCL were identified as key antigen proteins in iPSC, demonstrating promise for the development of peptide-based vaccines for both the prevention and treatment of CRC.

## Introduction

Colorectal cancer (CRC) is a globally prevalent malignancy with an increasing annual incidence 1. Metastasis, most commonly affecting the liver and lungs, is the primary cause of CRC-related mortality 2. Metastatic CRC remains incurable, with systemic drug therapies being the mainstay of treatment to prolong patient survival. Over the past two decades, advancements in chemotherapeutic and molecularly targeted agents have extended the median survival of patients with metastatic CRC from 12 to 24 months; however, the inevitable development of acquired resistance ultimately results in patient mortality 3-5. Current immunotherapies, such as immune checkpoint inhibitors (ICI), have demonstrated limited efficacy in CRC, particularly in cases with low microsatellite instability (MSI) or mismatch repair proficiency 6, 7. Given these challenges, innovative approaches such as induced pluripotent stem cell (iPSC)-based vaccines present a promising strategy for CRC prevention and treatment by potentially inducing robust and specific immune responses against tumor-associated antigens (TAAs).

The adaptive immune system has the ability to recognize and mount responses against non-mutated TAAs 8. Sipuleucel-T (Provenge^®^), a TAA-based therapeutic cancer vaccine approved by the Food and Drug Administration (FDA), exemplifies the clinical application of this approach 9. Studies have reported similarities in transcriptomic features and antigen expression between various tumor cells and embryonic stem cells (ESCs) 10. Immunization with ESCs has been shown to induce tumor rejection in mice 11, 12. Induced pluripotent stem cells (iPSCs) serve as an alternative to ESCs and exhibit similar gene expression patterns and surface marker profiles 13, 14. iPSCs have been reported to share gene expression profiles with cancer cells 15-17. Cluster analysis of RNA-Seq data from iPSC and cancer cell lines identified a set of upregulated genes, termed iPSC-cancer signature genes, which are highly expressed in pluripotent populations but minimally or not at all in somatic tissues 15. iPSCs share key hallmarks with cancer cells, including sustained proliferative signaling and replicative immortality 18, 19. This may be due to iPSC reprogramming, which is driven by introducing the four Yamanaka factors (Oct4, Sox2, c-Myc, and Klf4) into terminally differentiated somatic cells, partially recapitulating the process of somatic cell carcinogenesis. Notably, iPSCs have also been reported to share TAA profiles with cancer cells. These findings suggest that shared proteins between iPSCs and cancer cells encompass non-mutated TAAs capable of inducing antitumor immunity. Our previous study demonstrated that an iPSC-based vaccine effectively prevented tumor growth in various cancer mouse models, including melanoma, breast, lung, and pancreatic cancers 15, 20. However, whether an iPSC-based vaccine can induce effective antitumor immunity in CRC remains unclear. In addition, the underlying mechanisms and the therapeutic efficacy of the iPSC-based vaccine remain unclear. This study aimed to evaluate the prophylactic and therapeutic efficacy of the iPSC-based vaccine in CRC models.

In this study, we demonstrated that an iPSC-based vaccine exhibited both prophylactic and therapeutic efficacy in reducing tumor growth in CRC mouse models. Vaccination significantly increased CD8^+^ T cell infiltration within tumor regions, while T cell depletion abrogated the antitumor effects, underscoring the critical role of T cells in mediating these responses. Liquid chromatography-mass spectrometry (LC-MS) analysis combined with NetMHCpan-4.1 prediction server identified two iPSC-associated proteins, heterogeneous nuclear ribonucleoprotein U (HNRNPU) and nucleolin (NCL), as strong MHC I binders. Vaccination with cell lysates from CHO-K1 cells overexpressing HNRNPU or NCL, in combination with CpG, enhanced dendritic cell activation, induced antigen-specific CD8^+^ T cell cytotoxicity, and promoted the formation of central memory CD8^+^ T cells, collectively leading to significant tumor shrinkage in CRC models. These findings elucidate a potential mechanism of action for iPSC-based cancer vaccines and highlight their promise in advancing cancer immunotherapy.

## Materials and methods

### Animal model for CRC allografts

Young adult male C57BL/6J and BALB/c nude mice (6-8 weeks old) were purchased from the National Science Council Animal Center (Taiwan). The allograft mouse model procedure has been described previously 21. Briefly, each mouse was subcutaneously inoculated with 2 × 10^6^ MC38 CRC cells suspended in 50 μL of PBS and then mixed with 50 μL of Matrigel (CORNING, 354234, Corning, NY, USA). The cell mixture was subcutaneously inoculated into the right hind flank of nude mice. One week after tumor inoculation, the mice received the vaccine once per week for three consecutive weeks. The vaccines were administered subcutaneously near the lymph nodes, with the injection site rotated weekly. Tumor volume and body weight were monitored every 2-3 days, while bioluminescence imaging was performed weekly. The mice were sacrificed on day 21, and tissues were collected, fixed in 4% formaldehyde, and embedded in paraffin. Tissue sections (5-8 μm) were prepared on standard glass microscope slides, and hematoxylin and eosin (H&E) staining was performed by the National Taiwan University Laboratory Animal Center (NTULAC). Mice were treated in accordance with protocols approved by the Institutional Animal Care and Use Committee (IACUC) of the College of Medicine, National Taiwan University.

### iPSC vaccine preparation and immunization

The procedure for iPSC vaccine preparation was performed as previously described 15. For each mouse, 2 × 10⁶ SSEA-1-sorted syngeneic murine iPSCs were irradiated at 6,000 rads prior to injection. The irradiated iPSCs were suspended in 5 mM CpG ODN 1826 in PBS and loaded into 28G insulin syringes (Terumo, 443-828155, Tokyo, Japan). Mice were anesthetized with 2% isoflurane (Isothesia, Butler Schein) in 100% oxygen until the loss of the righting reflex. Immunization was carried out via subcutaneous injection of the vaccine into the flanks, with the injection site alternated weekly. Mice were monitored weekly for general health through gross examination, assessment of overall appearance, and weight measurements. The investigator analyzing tumor sizes and data from the adjuvant treatment experiment was blinded to the treatment groups.

### Analysis of RNA-Sequencing (RNA-Seq) data

Total RNA was extracted from tumor tissues using QIAzol reagent, and the quality of the extracted RNA was assessed using Trimmomatic software for quality control. RNA libraries were constructed, and sequencing was performed on the NovaSeq 6000 Sequencing System (Illumina Inc., San Diego, CA, USA). Gene expression levels were normalized using DESeq2 software, and expression analyses were conducted on the Biotools cloud platform (Bioconductor Software). Gene expression was quantified as transcripts per million (TPM) using RSEM, and log₂-transformed TPM values (log₂TPM) were used for statistical analyses 22.

### Isolation of mouse leukocytes from tumor tissues, spleens, and lymph nodes

Excised tumors (0.5 g) were collected and dissected into small fragments in a 60 mm Petri dish containing 3 mL Hank's Balanced Salt Solution (HBSS) (Thermo Fisher Scientific, 88284). The fragments were transferred to 10 cm dishes and digested with 10X Double Enzyme Mix (1 g Collagenase IV and 20,000 Units DNase IV in 100 mL HBSS) diluted in HBSS. Digestion was performed at 37 °C with shaking at 80 rpm for 90 minutes. The cell suspension was centrifuged at 100 × g for 10 minutes, and the supernatants were filtered through a 40 μm cell strainer. The filtered cells were centrifuged at 400 × g for 3 minutes to collect the leukocytes. Red blood cells were lysed using ACK Lysing Buffer (Lonza, BP10-548E, Basel, Switzerland), and the remaining cells were resuspended in FACS buffer (0.2% bovine serum albumin in PBS). For isolating leukocytes from tumor-draining lymph nodes and spleens, the tissues were pressed through 30 μm MACS SmartStrainers (Miltenyi Biotec, 130-098-458, Bergisch Gladbach, Germany) to obtain single-cell suspensions.

### Extraction of splenic CD8^+^ T cells

CD8^+^ T cells were isolated from the spleens of C57BL/6J mice. Splenocytes were prepared by pressing spleens through 30 μm MACS SmartStrainers (Miltenyi Biotec). Red blood cells were lysed using ACK Lysing Buffer, and the remaining cells were collected by centrifugation. CD8^+^ T cells were purified using the CD8a^+^ T Cell Isolation Kit (Miltenyi Biotec, 130-104-075) following the manufacturer's protocol. Isolated CD8^+^ T cells were cultured in RPMI 1640 medium (GIBCO, 11875085, Waltham, MA, USA) supplemented with 10% fetal bovine serum (FBS; Thermo Fisher Scientific, A5256701, Waltham, MA, USA), 50 μM 2-mercaptoethanol (Thermo Fisher Scientific, 21985023), and 1% antibiotic-antimycotic solution (Gemini Bio-Products, 400-101-100, Sacramento, CA, USA).

### Generation of mouse bone marrow-derived dendritic cells (BMDCs)

Mouse bone marrow cells were harvested from femurs and tibiae by flushing with PBS and dispersed using an 18G needle. The cells were centrifuged at 1300 rpm for 5 minutes, and red blood cells were lysed with ACK lysis buffer for 1 minute before being diluted in 25 mL PBS. The cell suspension was filtered through a 30 µm SmartStrainer and centrifuged again at 1300 rpm for 5 minutes. Bone marrow cells (10⁶ cells per well) were cultured in RPMI medium supplemented with 50 µM 2-mercaptoethanol and 100 ng/mL Flt3L in a 24-well plate. On day 3, 1 mL of fresh medium containing Flt3L was added to each well. By day 6, BMDCs were fully differentiated, with approximately 70% of cells expressing CD11c as confirmed by flow cytometry.

### LC-MS analysis and neoantigen discovery

LC-MS analysis was conducted at the Metabolomics Core Laboratory, National Taiwan University Center of Genomics and Precision Medicine (Taipei, Taiwan). Equal amounts of iPSCs, MC38 CRC cells, and MEFs (10^7^ cells) were collected and washed with PBS, followed by centrifugation at 200 × g for 5 minutes to remove the supernatant. The cell pellets were resuspended in 500 μL of ice-cold methanol, vortexed using a GenoGrinder at 1000 rpm for 2 minutes, and incubated on ice for 5 minutes. Next, 500 μL of ice-cold water was added, followed by another round of vortexing and ice incubation. The samples were centrifuged at 15,000 × g for 5 minutes, and the supernatants were transferred to fresh tubes and dried overnight. The dried residues were stored at -80 °C until further analysis using the 6540 Accurate-Mass Quadrupole Time-of-Flight (Q-TOF) LC-MS system (Agilent, Santa Clara, CA, USA). For in silico prediction, the binding affinities of 8- to 11-mer peptides derived from iPSC-expressed antigens to MHC class I or HLA class I were evaluated using NetMHCcons 1.1. Peptides with predicted binding affinity values (IC50 < 500 nM) were considered potential neoantigens.

### Establishment of protein-overexpressing CHO-K1

CHO-K1 cells were seeded in 6-well plates and allowed to adhere overnight. Plasmids (pEGFP-N1-mNCL, pEGFP-N1-mSTMN1, pEGFP-N1-mHNRNPU, pEGFP-N1-mMARCKSL1, pEGFP-N1-mNASP) and Lipofectamine 2000 (Thermo Fisher Scientific, 11668019) were diluted separately in Opti-MEM (Thermo Fisher Scientific, 31985062) and incubated at room temperature for 5 minutes. The diluted plasmids and Lipofectamine 2000 were then gently mixed and incubated for 20 minutes at room temperature to form DNA-liposome complexes. The complexes were added dropwise to the cells and incubated at 37 °C for 6 hours. Afterward, the medium was replaced with fresh culture medium, and the cells were further incubated for 48 hours. Subsequently, the cells were harvested and subjected to fluorescence-activated cell sorting (FACS) to isolate GFP-expressing cells. The sorted GFP-positive cells were cultured in medium containing G418 (Invivogen, ant-gn-1, San Diego, CA, USA) for selection.

### Western blot analysis

Sample protein was extracted by lysis buffer containing 100X protease and phosphatase inhibitors, and quantified using Pierce Bradford Protein Assay Kit (Thermo Fisher Scientific, 23200). The concentration of protein was measured by Enzyme-linked Immuno-sorbent Assay (ELISA) reader. Cells were lysed on ice. Total cell lysates were centrifuged at 13,000 rpm for 15 min at 4 °C and then subjected to SDS PAGE using adequate percentage polyacrylamide gels. Immunoblotting was performed using specific antibodies to evaluate the expression of different proteins (**Table S1**).

### Scanning Electron Microscopy (SEM) imaging

Glass coverslips were coated with 100 μg/mL poly-L-lysine solution (Elabscience, PB180523, Houston, TX, USA) in ultra-pure water and incubated at 4 °C overnight. BMDCs (4 × 10⁵) were seeded onto the coated coverslips and activated with CpG and iPSC cell lysates for 18 hours. After cell adhesion, the coverslips were washed three times with PBS and fixed in 2.5% glutaraldehyde overnight at 4 °C. The fixed cells were washed three times with 0.1 M phosphate buffer and post-fixed in 1% osmium tetroxide for 1 hour. Subsequently, the specimens were rinsed in 0.1 M phosphate buffer and dehydrated through a graded ethanol series (30%, 50%, 70%, 85%, 90%, 95%, and 100%) for 10 minutes at each concentration. The specimens were then washed twice with 100% acetone for 10 minutes each. Dehydrated samples were subjected to critical point drying and coated with a platinum layer using a sputter coater. Imaging was performed using a Scanning Electron Microscope (FlexSEM 1000 II, HITACHI, Chiyoda City, Japan).

### Transwell migration assay

BMDCs were stimulated with 0.075 nM CpG and cell lysates for 6 hours at a DC-to-cell lysate ratio of 1:3. Matured BMDCs (5 × 10⁴) were co-cultured with splenic CD8^+^ T cells (5 × 10⁵), which were labeled with the CellTracker Fluorescent Probe CMRA (Thermo Fisher Scientific, C34551), for 18 hours. MC38 cells (5 × 10⁴) were seeded into the lower chambers of a 24-well transwell plate and allowed to adhere overnight. The activated CD8^+^ T cells were then seeded into the upper chambers. Following a 3-hour incubation at 37 °C, the upper chambers were removed, and the migrated cells in the lower chambers were imaged using the EVOS M7000 Imaging System (Thermo Fisher Scientific).

### CD8^+^ T cell cytotoxicity assay

CD8^+^ T cell cytotoxicity assay: BMDCs were stimulated with 0.075 nM CpG and cell lysates (DC: cell lysate = 1:3) for 6 hours. Matured BMDCs (5 × 10⁴) were then co-cultured with splenic CD8^+^ T cells (5× 10⁵) for 18 hours. Next, MC38 cells (2.5 × 10⁴) were seeded in 24-well plates and allowed to adhere overnight. Activated CD8^+^ T cells were then co-cultured with MC38 cells for 24 hours. After incubation at 37°C for 24 hours, the cells were harvested for flow cytometry analysis, and the supernatants were collected for ELISA.

### Statistical analysis

Prism software V.5.0.0 (GraphPad Software Inc., La Jolla, CA, USA) was used for statistical analyses, including one-way and two-way analysis of variance (ANOVA). Statistical significance was defined as p < 0.05 (*), p < 0.01 (**), and p < 0.001 (***). Data shown are representative of at least three independent experiments. Quantitative data are presented as the mean ± standard deviation (SD) values.

### Additional methods

Detailed methodology is described in the Supplementary materials and methods.

## Results

### Prophylactic effects of the iPSC-based vaccine on colorectal tumor growth in mouse models

**Figure 1:**
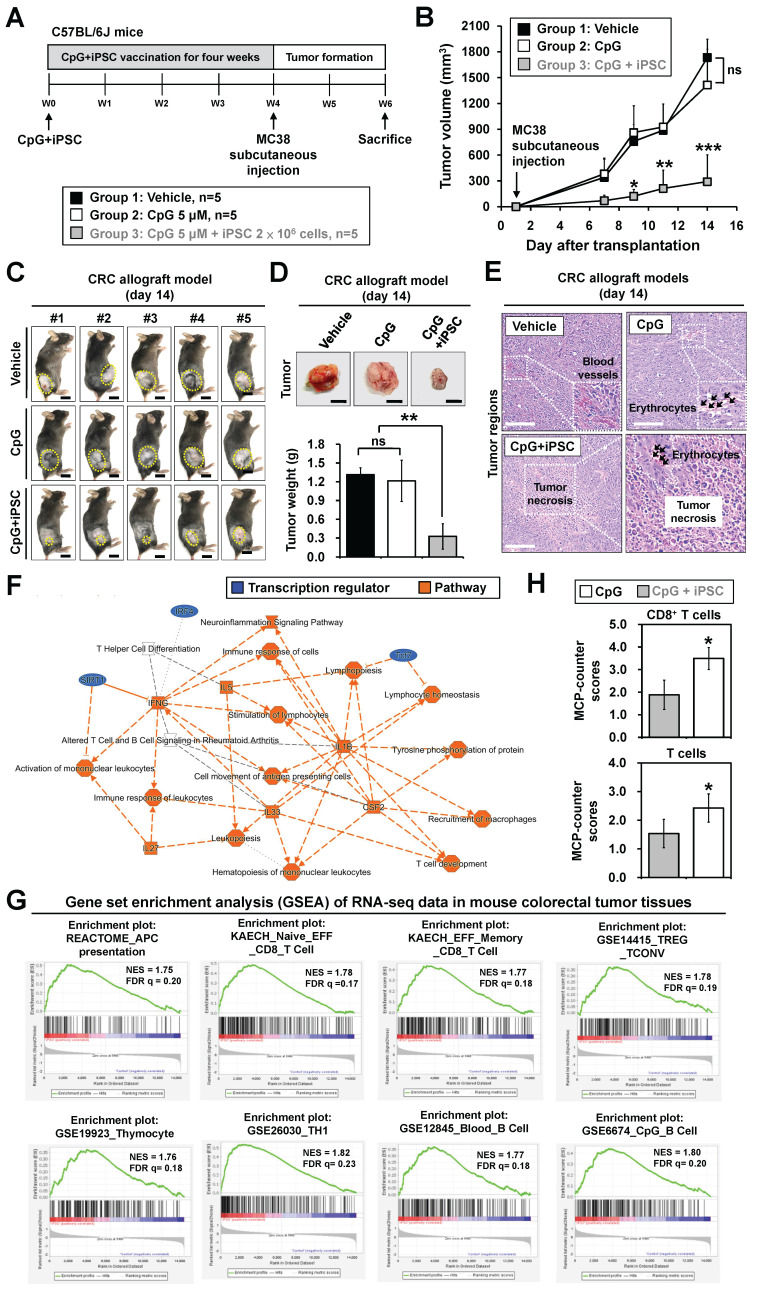
** Prophylactic effect of the iPSC-based vaccine in colorectal cancer allograft mouse models.** Male C57BL/6 mice were vaccinated for 4 weeks with PBS (vehicle), CpG (5 µM), or CpG plus iPSCs (2 × 10⁶). MC38 cells (2 × 10⁶) were then subcutaneously implanted into the lower back, and tumor growth was measured every two days for two weeks. **(A)** Schematic overview of the experimental design. **(B)** Tumor volume was calculated using the formula: V = 0.5 × (length of the longest diameter) × (length of the shortest diameter)^2^. Two-way ANOVA was used to define the p-value. *p < 0.05; **p < 0.01; ***p < 0.001; ns, no significance.** (C)** Representative images of tumor burden. Yellow circles indicate tumor regions. Scale bar: 1 cm.** (D)** Gross images of allograft tumors (upper panel) and excised tumor weights (lower panel) at the end of the experiment. Scale bar: 1 cm. **p < 0.01; ns, no significance.** (E)** Tumor sections were counterstained with H&E, and a high-magnification image of the area in the white box is shown. Scale bars: 250 μm.** (F-H)** Bulk RNA-Sequencing (RNA-Seq) analysis of tumor tissues from colorectal cancer allograft mouse models.** (F)** Ingenuity Pathway Analysis (IPA) identified immune-related molecular networks and genes with >2-fold expression changes.** (G)** Gene Set Enrichment Analysis (GSEA) results show enriched immune response pathways in MC38 tumor tissues. NES, normalized enrichment score; FDR, false discovery rate (q-value). **(H)** Murine Microenvironment Cell Population (MCP)-Counter analysis reveals differences in immune and stromal cell populations between CpG and CpG + iPSC groups. MCP-Counter scores of T cell populations, highlighting differences between CpG and CpG + iPSC groups. *p < 0.05.

To study whether iPSCs can stimulate the immune system to prevent colorectal cancer, MC38 allograft mouse models were used (**Figure 1A**). Mice were subcutaneously injected once a week for 4 weeks with (1) phosphate-buffered saline (PBS) as vehicle control, (2) CpG alone, or (3) the combination of CpG and iPSCs (C + I), with 5 mice per group. Afterward, the mice were inoculated at a separate site with MC38, a well-established murine colon adenocarcinoma cell line (**Figure 1A**). By day 14 after tumor inoculation, tumor volume in the C + I-vaccinated mice was significantly lower compared to the PBS and CpG groups (**Figure 1B-D** and **S1A**). Interestingly, we observed that three mice showed a complete response in the C + I group (**Figure S1A-C**). No significant difference in tumor size was observed between the PBS and CpG groups. Histological examination of tumor tissues revealed that subcutaneous tumors formed by MC38 CRC cells exhibited typical malignant features, such as cellular atypia and pathological mitotic figures, while the C + I group exhibited tumor necrosis within the tumor regions (**Figure 1E** and** S1C**). Erythrocyte aggregation was observed in both the CpG and C + I groups. Erythrocyte aggregation plays a crucial role in circulation and may influence blood flow and inflammatory responses 23. In tumor tissues, it may be associated with hypoxic regions and inflammation induced by tumor cells. In addition, spleen weight was increased in both the CpG alone and C + I groups (**Figure S1D**). These results demonstrate the effectiveness and tumor-preventive effects of the iPSC-based cancer vaccine in CRC.

### The iPSC-based vaccine activates T cell populations in colorectal cancer mouse models

To investigate the mechanism underlying the tumor-preventive effects of the iPSC-based vaccine, we next performed bulk RNA-Seq on vaccinated mice. Tumor tissues were harvested from the CpG and C + I groups (**Figure S1E**-**F**). Ingenuity Pathway Analysis (IPA) is a commercial bioinformatics software that allows canonical pathway analysis of RNA-Seq data against a manually curated pathway database 24. IPA analysis revealed that the upregulated genes in the C + I group were involved in immune responses, including T cell differentiation, lymphocyte stimulation, and lymphocyte homeostasis (**Figure 1F**). Gene Set Enrichment Analysis (GSEA) is a computational method used to identify biological insights within gene expression data 25. GSEA results showed that the enriched gene sets in the C + I group included APC presentation, CD8 T cell, TH1, and B cell-associated gene sets (**Figure 1G**). The murine Microenvironment Cell Populations-counter (mMCP-counter) method, based on highly specific transcriptomic markers, accurately quantifies immune and stromal murine cell populations 26. The heatmap of MCP-counter analysis showed that the proportion of T cells, CD8 T cells, memory B cells, and monocytes/macrophages in the C + I group were significantly higher than in the CpG group (**Figure S1G**). Quantification of mMCP-counter results showed increased populations of T cells and CD8 T cells in the C + I group (**Figure 1H**). These results indicate that the combination of CpG and iPSCs exerts a synergistic effect in activating the immune system and inducing tumor-preventive effects in CRC.

### Depletion of T cells abrogates tumor-preventive effects of the iPSC-based vaccine in colorectal cancer

**Figure 2:**
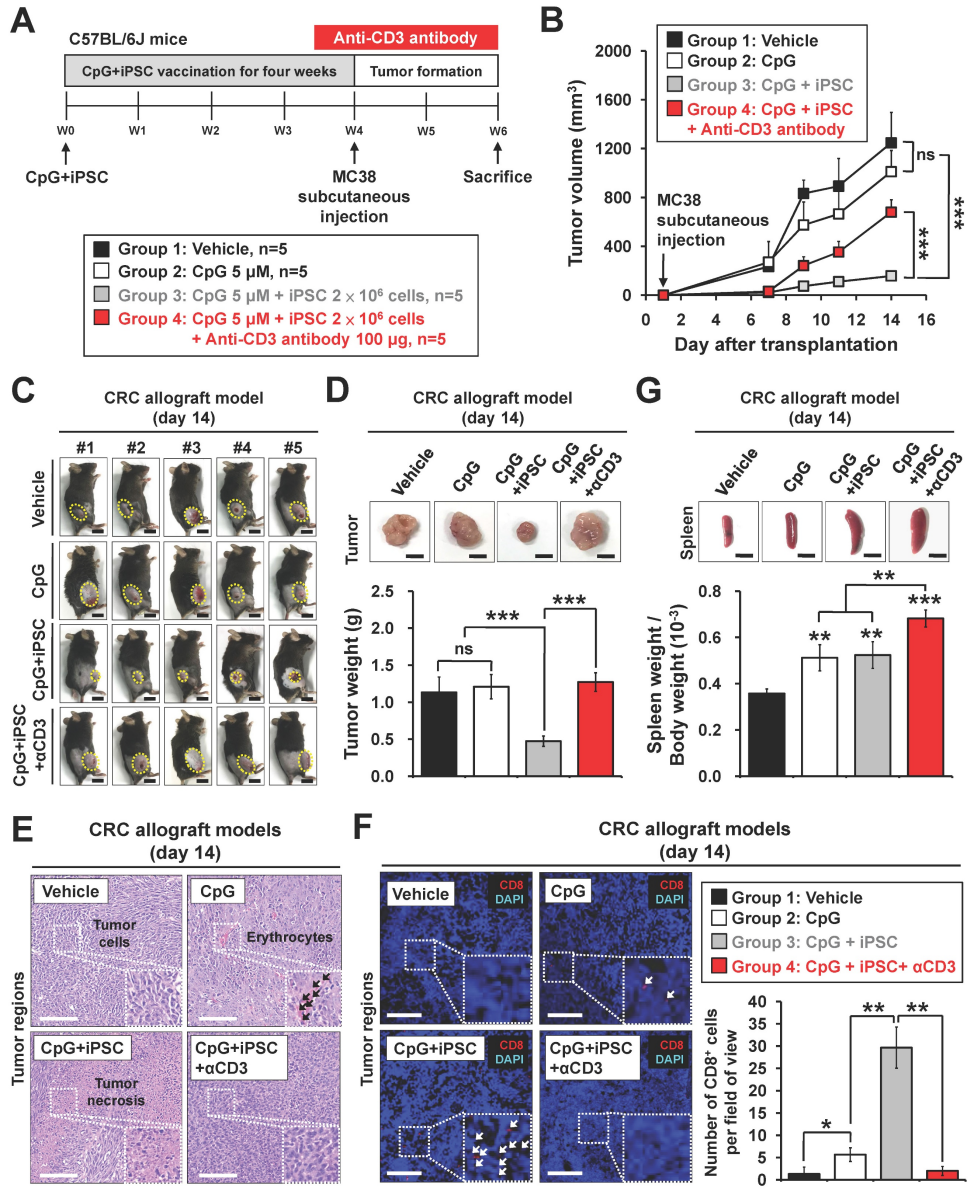
** Depletion of CD3^+^ T cells abolishes iPSC-mediated prophylactic effects in colorectal cancer allograft mouse models.** Male C57BL/6 mice were vaccinated for 4 weeks with vehicle (PBS), CpG (5 µM), or CpG plus iPSCs (2 × 10⁶). In the CpG + iPSCs group, anti-CD3 antibody (50 μg, intraperitoneally) was administered every two days for two weeks post-vaccination. MC38 cells (2 × 10⁶) were then subcutaneously implanted into the lower back of the mice, and tumor growth was measured every two days for two weeks.** (A)** Schematic overview of the experimental design.** (B)** Tumor volume was calculated using the formula: V = 0.5 × (length of the longest diameter) × (length of the shortest diameter)^2^. Two-way ANOVA was used to define the p-value. ***p < 0.001; ns, no significance.** (C)** Images of the tumor burden in each group. The yellow circle indicates the tumor region. Scale bar: 1 cm. **(D)** Gross images of allograft tumors (upper panel) and excised tumor weights (lower panel) at the end of the experiment. Scale bar: 1 cm. ***p < 0.001; ns, no significance.** (E)** Tumor sections were counterstained with H&E, and a high-magnification image of the area in the white box is shown. Scale bars: 250 μm.** (F)** Immunofluorescence analysis of CD8^+^ T cells (red fluorescence) in tumor sections. Nuclei were counterstained with DAPI (blue fluorescence). High-magnification images of boxed areas show CD8^+^ cells (white arrowheads). Scale bars: 200 μm. Quantification of CD8^+^ cells in tumor regions is shown. *p < 0.05; **p < 0.01.** (G)** Gross images of spleens (upper panel) and spleen weights (lower panel) at the end of the experiment. Scale bar: 1 cm. ns, no significance. **p < 0.01; ***p < 0.001.

To investigate whether activation of T cells plays a role in iPSC-mediated vaccination, anti-CD3 antibody was used in MC38 mouse models (**Figure 2A**). The C + I treatment significantly reduced MC38 tumor growth; however, this effect was abolished by anti-CD3 antibody treatment (**Figure 2B-D**). Interestingly, the C + I + αCD3 group exhibited the largest tumor weights. Histological examination of tumor tissues revealed that the C + I group showed tumor necrosis and CD8^+^ T cell infiltration within the tumor regions, while these effects were abolished by T cell depletion (**Figure 2E**-**F**). In addition, spleen weight was increased in both the C + I and C + I + αCD3 groups (**Figure 2G**).

BALB/c nude mice lack a thymus due to a mutation in the FOXN1 gene, resulting in the absence of T cell production 27. We also investigated the tumor-preventive effects of the iPSC-based vaccine in nude mouse models (**Figure S2A**). Similar results were observed in these nude mouse models. MC38 tumors showed significant growth in mice treated with PBS or CpG alone by day 14 after tumor inoculation, while the C + I treatment did not alter tumor volume or weight in nude mice (**Figure S2B-D**). Histological examination of tumor tissues further confirmed these findings (**Figure S2E**-**F**). Additionally, no significant change in spleen weight was observed between groups (**Figure S2G**). These results indicate that T cell populations play a critical role in the tumor-preventive effects of the combination of iPSC + CpG in CRC.

### Therapeutic effects of the iPSC-based vaccine on colorectal tumor growth in mouse models

**Figure 3:**
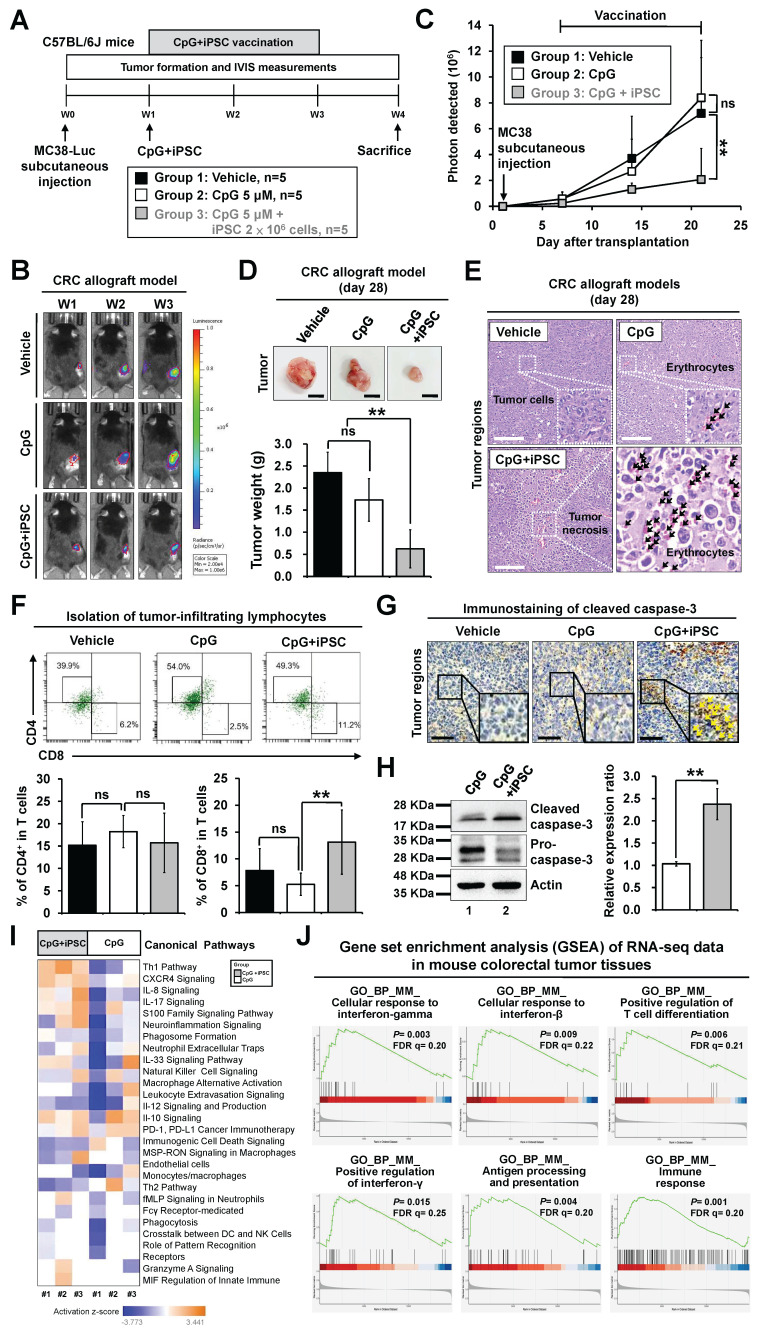
** Therapeutic effects of the induced pluripotent stem cell-based vaccine in colorectal cancer allograft mouse models.** Male C57BL/6 mice were vaccinated for 4 weeks with vehicle (PBS), CpG (5 µM), or CpG plus iPSCs (2 × 10⁶). MC38-Luciferase-expressing cells (2 × 10^5^) were then subcutaneously implanted into the lower back. Endotoxin-free luciferase substrate was administered weekly, and luminescence was detected using the IVIS imaging system (Xenogen). Tumor growth was measured every two days for two weeks. **(A)** Schematic overview of the experimental design.** (B and C)** Therapeutic effects of the iPSC-based vaccine on MC38 tumor growth were assessed using the IVIS imaging system.** (B)** Weekly luciferase activity was measured with the IVIS imaging system over three weeks, and luminescence intensity is represented using a blue-to-red spectrum.** (C)** Synchronized images were quantified weekly for three weeks. Two-way ANOVA was used to define the p-value. **p < 0.01; ns, no significance.** (D)** Gross images of allograft tumors (upper panel) and excised tumor weights (lower panel) at the end of the experiment. Scale bar: 1 cm. **p < 0.01; ns, no significance. **(E)** Tumor sections were counterstained with H&E, and a high-magnification image of the area in the white box is shown. Scale bars: 250 μm.** (F)** Flow cytometry plots showing CD4^+^ T cells (CD45^+^CD3^+^CD4^+^) and CD8^+^ T cells (CD45^+^CD3^+^CD8^+^) in tumor regions (upper panel). Percentages of CD4^+^ and CD8^+^ T cells in the CpG and CpG + iPSC groups are shown (lower panel).** (G)** Immunostaining of cleaved caspase-3 in tumor sections, with high-magnification images of boxed areas. Scale bars: 250 μm.** (H)** Western blot analysis of cleaved caspase-3 expression in tumor tissues (left). Protein levels were quantified using ImageJ (right). **p < 0.01. **(I)** Heatmap of IPA analysis displayed the signaling pathways between CpG and CpG + iPSC groups.** (J)** Gene Set Enrichment Analysis (GSEA) enrichment analysis of immune response in mouse MC38 tumor tissues. NES, normalized enrichment score; false discovery rate, FDR (q-value).

Recent therapeutic cancer vaccines have shown promise by eliciting de novo T cell responses targeting tumor antigens, including tumor-associated and tumor-specific antigens 28. We further investigated the therapeutic effect of the iPSC-based vaccine in MC38-Luciferase-expressing CRC mouse models and monitored tumor progression using the IVIS imaging system (**Figure 3A**). The results demonstrated that C + I treatment inhibited tumor growth in the CRC mouse models (**Figure 3B**-**C**). A reduction in tumor weight was also observed in the C + I group (**Figure 3D**). Histological examination of tumor tissues revealed that the C + I treatment induced tumor necrosis in the tumor regions (**Figure 3E**). An increase in tumor-infiltrating CD8^+^ T cells was observed in the tumor regions of the C + I mice (**Figure 3F**). Immunohistochemistry and Western blotting analysis showed that C + I treatment induced cleaved caspase-3 in tumors (**Figure 3G**-**H**).

To investigate the mechanism underlying the tumor-preventive effects of the iPSC-based vaccine, bulk RNA-Seq was performed on vaccinated mice. Tumor tissues were harvested from the CpG and C + I groups (**Figure S3A**-**B**). IPA analysis revealed that the upregulated genes in the C + I group were involved in immune responses, including cellular response to interferon-γ, T cell differentiation, antigen processing and presentation, and the Th1 pathway (**Figure 3I** and **S3C**). GSEA results showed that enriched gene sets in the C + I group included response to interferon-γ, T cell differentiation, antigen processing and presentation, and immune response-associated pathways (**Figure 3J**). The heatmap of MCP-counter analysis showed that the proportion of NK cells, T cells, CD8 T cells, memory B cells, and monocytes/macrophages were significantly higher in the C + I group compared to the CpG group (**Figure S3D**). These findings indicate that the combination of CpG and iPSCs exerts an anti-tumor therapeutic effect in CRC through activation of the immune system.

### The iPSC-based vaccine also showed therapeutic effects on colitis-associated CRC and metastatic CRC in mouse models

**Figure 4:**
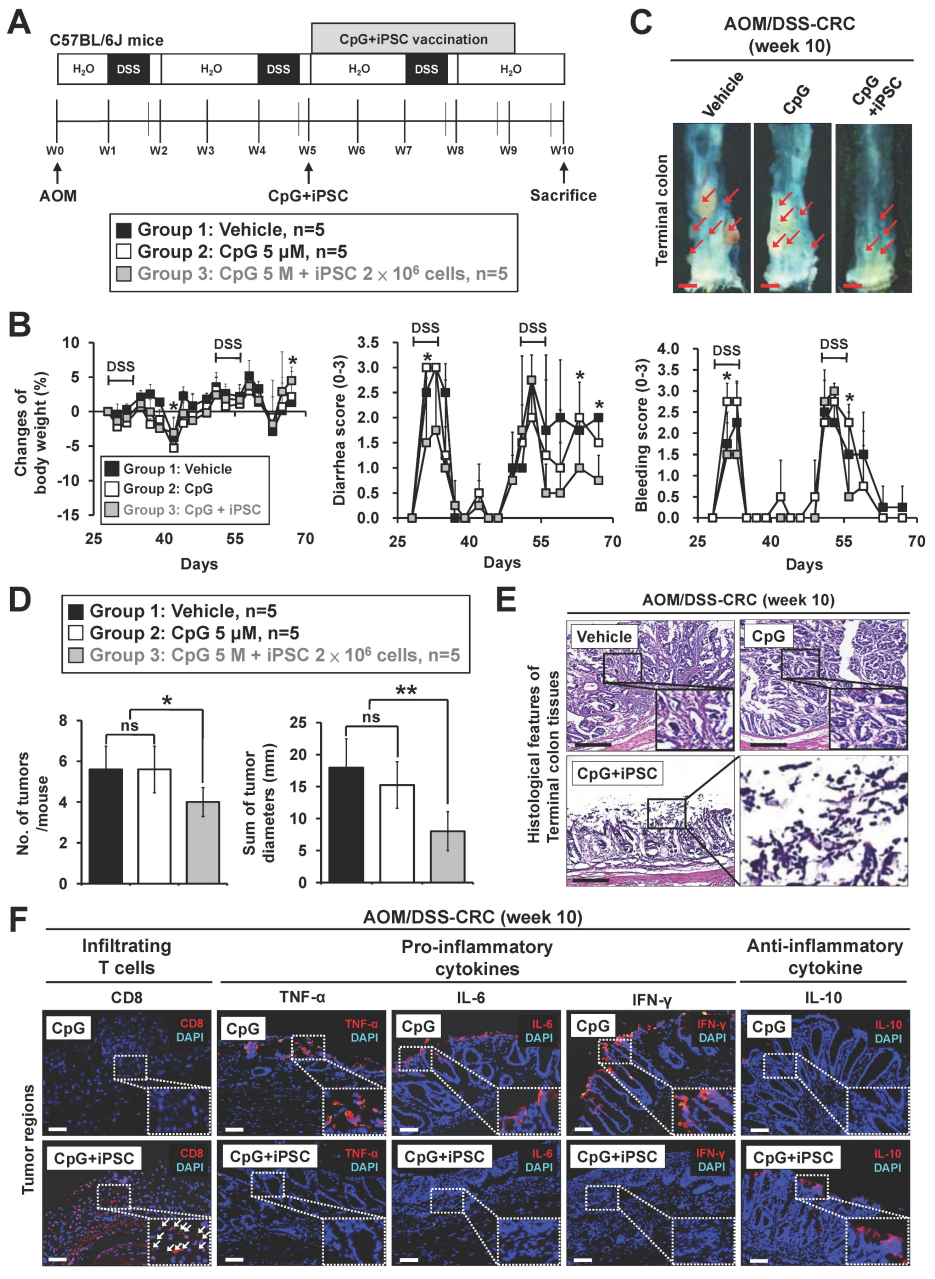
** Therapeutic effects of the induced pluripotent stem cell-based vaccine in AOM/DSS-indcued colorectal cancer mouse models.** Colitis-associated CRC was induced in B6 mice by AOM/DSS treatment (n = 15). AOM/DSS-treated mice were randomly divided into three groups. Mice were vaccinated at week 5 for 4 weeks with vehicle (PBS), CpG (5 µM), or CpG plus iPSCs (2 × 10^6^ cells). **(A)** Schematic overview of the experimental design. **(B)** Changes in body weight (left panel), clinical diarrhea scores (middle panel), and clinical bleeding scores (right panel) during treatment are shown. Two-way ANOVA was used to define the p-value. *p < 0.05 versus CpG. **(C)** The therapeutic effect of iPSC-based vaccine on AOM/DSS-induced CRC. Gross images of terminal colons are shown, and the red arrowhead indicates macroscopic polyps. Scale bars: 1 mm. **(D)** The number and size of tumors were plotted. *p < 0.05; **p < 0.01; ns, no significance. **(E)** Colon sections from the mouse models were counterstained with H&E, and a high-magnification image of the area in the black box is shown. Scale bars: 250 μm. **(F)** Colon sections from AOM/DSS-treated mice were immunostained with specific antibodies. High-magnification images of the areas in the white boxes are shown. The white arrowhead indicates the CD8^+^ cells. Scale bar: 250 μm.

Chronic non-resolving inflammatory bowel disease (IBD), such as Crohn's disease and ulcerative colitis, is a predisposing factor for CRC, increasing the cumulative risk of CRC development by up to 5- to 8-fold in IBD patients 29. Although IBD-associated CRC is rare, constituting only 2%-3% of all CRC cases, it represents a significant risk factor and is classified as colitis-associated cancer 30. The azoxymethane/dextran sulfate sodium (AOM/DSS) mouse model is the most commonly used model to study colitis-associated cancer 31. We further evaluated the therapeutic effect of the iPSC-based vaccine in AOM/DSS-induced CRC mouse models (**Figure 4A**). Symptom parameters, including body weight loss, diarrhea, and rectal bleeding, were observed after AOM/DSS treatment. The C + I treatment significantly reduced the occurrence of these symptoms (**Figure 4B**). While the CpG-alone group showed no effect on the number and size of colon tumors, these tumors were markedly reduced in the C + I group (**Figure 4C**-**D**). Histological examination of colon tissues revealed that the C + I treatment induced tumor necrosis within the tumor regions (**Figure 4E**). An increase in tumor-infiltrating CD8^+^ T cells was observed in the tumor regions of the C + I-treated mice (**Figure 4F**). Interestingly, C + I treatment also downregulated proinflammatory cytokines and upregulated anti-inflammatory cytokines (**Figure 4F**). These findings demonstrate that the combination of CpG and iPSCs exhibits therapeutic efficacy in colitis-associated CRC in mouse models.

Metastasis is a leading cause of mortality in CRC, with the liver and lungs being the most common metastatic sites. 32 To evaluate the anti-metastatic activity of the combination of CpG and iPSCs, MC38 CRC cells were injected into the spleens of C57BL/6J mice (**Figure S4A**). By day 21, liver tumor nodules were observed. While CpG alone had no effect on the number of liver tumor nodules, the C + I treatment significantly reduced the number of nodules in mice (**Figure S4B**-**C**). Histological examination of liver tissues further confirmed these findings (**Figure S4D**). Immunohistochemistry analysis revealed that C + I treatment induced cleaved caspase-3 expression in tumor nodules (**Figure S4E**). These results demonstrate that the combination of CpG and iPSCs provides therapeutic efficacy against CRC liver metastases in mouse models.

### Discovery of potential neoantigen proteins in the iPSC-based vaccine

**Figure 5:**
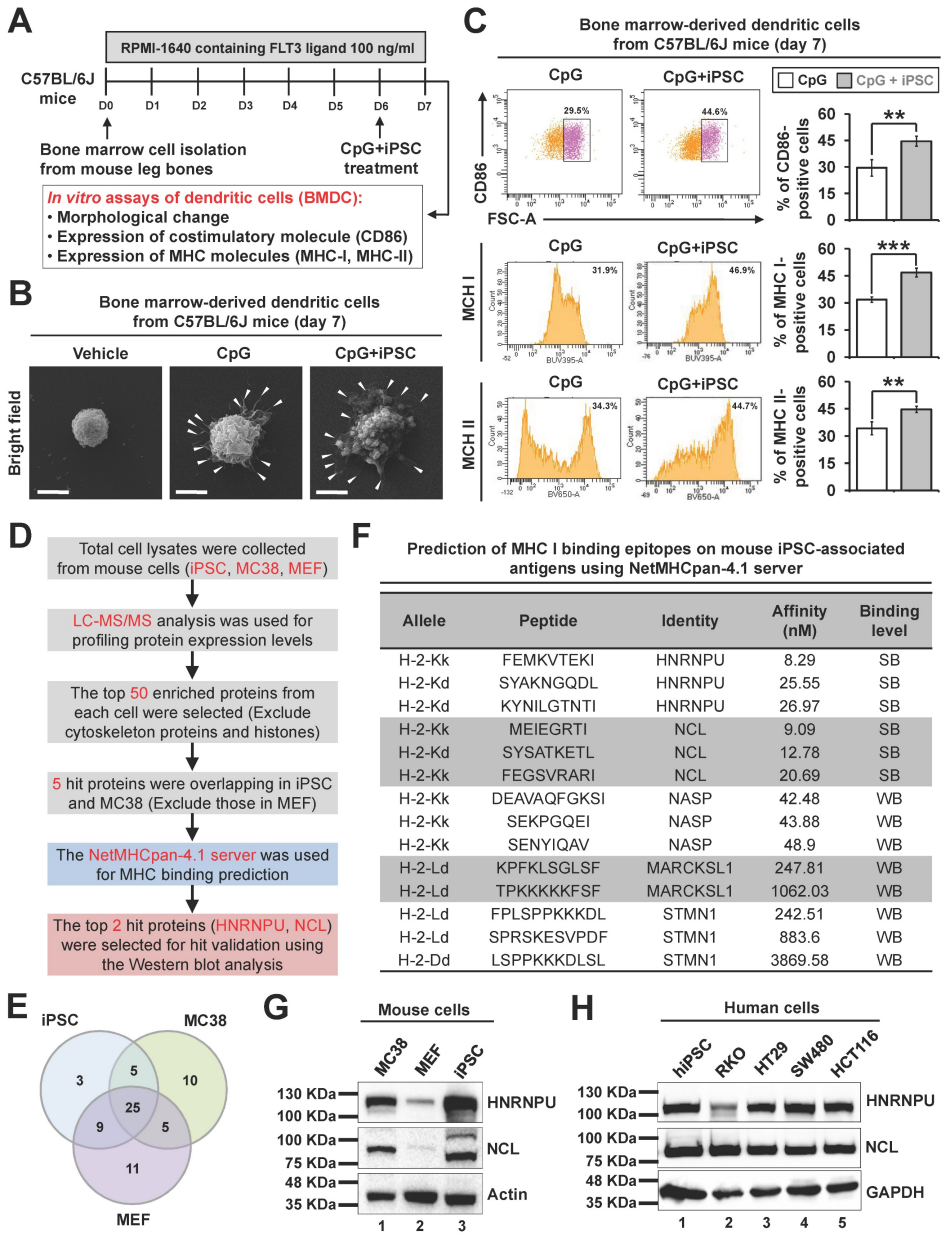
** Identification of potential antigens in iPSCs and colorectal cancer cells using LC-MS analysis. (A-C)** Effects of iPSC lysates on the immune response of mouse dendritic cells.** (A)** Protocol to isolate and analyze mouse bone marrow-derived dendritic cells (BMDCs) and schematic overview of the experimental design. **(B)** Morphological changes in BMDCs after CpG or CpG plus iPSC treatment, observed via transmission electron microscopy (TEM). White arrowheads indicate dendrites capturing cell lysates. Scale bars: 3 μm.** (C)** Representative flow cytometry plots showing surface marker expression on BMDCs. Percentages of marker expression after 24-hour stimulation with CpG or CpG plus iPSC were analyzed. **p < 0.01; ***p < 0.001. **(D and E)** Identification of potential antigens in iPSCs and CRC cells using LC-MS analysis.** (D)** Workflow for screening potential antigens via LC-MS analysis.** (E)** Venn diagram displaying the overlap of the top 50 enriched proteins identified in iPSCs and CRC cells.** (F)** Prediction of MHC I binding epitopes on potential antigens using the NetMHCpan-4.1 server (https://services.healthtech.dtu.dk/services/NetMHCpan-4.1/). Strong binders (SB) and weak binders (WB) were classified based on a predefined cutoff of 30 nM binding affinity.** (G)** Validation of two potential antigen proteins in iPSCs, MC38 CRC cells, and mouse embryonic fibroblasts (MEF) by Western blotting using specific antibodies.** (H)** Validation of the potential antigen proteins in four human CRC cell lines and human iPSCs by Western blotting using specific antibodies.

Dendritic cells are the most potent antigen-presenting cells and play a crucial role in tumor immunotherapies by inducing CD8^+^ T cell immunity 33. They uptake antigens and pathogens, generate MHC-peptide complexes, migrate from the sites of antigen acquisition to secondary lymphoid organs, and ultimately interact with and stimulate T lymphocytes. Whole tumor cell lysates have been utilized as tumor antigens in cancer vaccine development by enabling dendritic cells to present tumor antigens 34, 35. Given that the iPSC-based vaccine induces anti-tumor immunity through CD8^+^ T cell activation, we further explored whether dendritic cells can present antigens derived from iPSC whole cell lysates using mouse bone marrow-derived dendritic cell (BMDC) models (**Figure 5A**). The dendrites of dendritic cells are key structures for antigen uptake and presentation to T cells. Our results demonstrated that both the CpG group and the C + I group stimulated dendritic growth in mouse BMDCs (**Figure 5B**). Flow cytometry analysis revealed that C + I treatment significantly increased the expression of surface markers CD86, MHC I, and MHC II on BMDCs (**Figure 5C**). These findings suggest that dendritic cells play a pivotal role in presenting antigens derived from iPSC.

To identify potential neoantigen proteins within iPSC and CRC cells, we combined liquid chromatography-mass spectrometry (LC-MS) and in silico predictions (**Figure 5D**). Our analysis revealed five overlapping proteins between iPSC and MC38 CRC cells, excluding those expressed in MEF (**Figure 5E**). The NetMHCpan-4.1 server predicts peptide binding to any MHC I molecule based on artificial neural networks 36. We identified a strong binding affinity between MHC I and peptides derived from heterogeneous nuclear ribonucleoprotein U (HNRNPU) and nucleolin (NCL). Conversely, nuclear autoantigenic sperm protein (NASP), myristoylated alanine-rich C-kinase substrate-like 1 (MARCKSL1), and stathmin (STMN1) exhibited weaker binding affinities among the eight identified peptides with lengths of 7-11 amino acids (**Figure 5F**). Western blot analysis confirmed the expression of HNRNPU and NCL in both mouse iPSC and MC38 CRC cells but not in normal mouse embryonic fibroblasts (**Figure 5G**). Furthermore, HNRNPU and NCL were also expressed in human iPSC and various human CRC cell lines (**Figure 5H**). These findings suggest that HNRNPU and NCL are potential neoantigen proteins shared by iPSC and CRC cells, making them promising targets for immunotherapy.

### Evaluation and validation of *in vitro* immune responses induced by candidate neoantigen proteins

**Figure 6:**
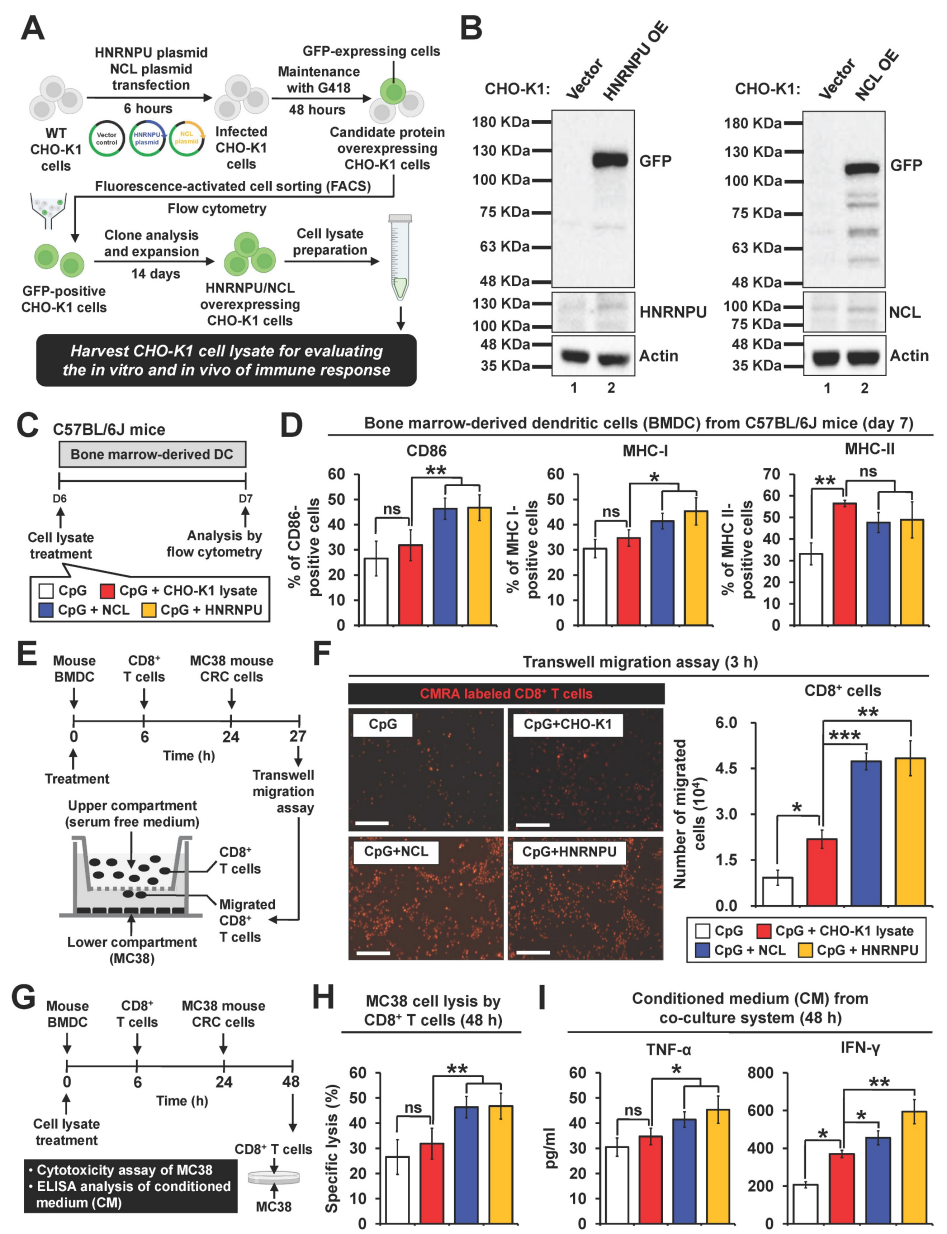
** Validation of immunogenicity of two candidate antigens in mouse dendritic cells. (A and B)** Development of stable CHO-K1 cell lines overexpressing HNRNPU or NCL. Briefly, plasmids expressing HNRNPU or NCL were transfected into CHO-K1 cells for 6 hours. Cells were treated with G418 (0.5 μg/ml) for 2 days. Afterward, cells were purified by fluorescence-activated cell sorting (FACS) using flow cytometry. Single clones were picked according to GFP expression and expanded to generate monoclonal cell colonies. **(A)** Schematic overview of the experimental design.** (B)** Validation of antigen protein expression in CHO-K1 cells using Western blot analysis with specific antibodies. **(C and D)** Antigen-presenting ability of BMDCs stimulated by HNRNPU and NCL. The ratio of mouse BMDCs and cell lysates is 1:3. **(C)** Schematic overview of the experimental design. **(D)** Percentages of surface marker expression on BMDCs after 24-hour stimulation with CpG or CpG plus antigen proteins. *p < 0.05; **p < 0.01; ***p < 0.001; ns, no significance.** (E and F)** Migration of CD8⁺ T cells toward CRC cells induced by HNRNPU and NCL. Mouse BMDCs were stimulated with antigens for 6 hours and co-cultured with CD8⁺ T cells at a 1:10 ratio for 18 hours. Activated CD8⁺ T cells were placed in the upper chamber of a transwell, with MC38 cells in the lower chamber. After 3 hours of incubation, CD8⁺ T cell migration was assessed. **(E)** Schematic overview of the experimental design.** (F)** Representative fluorescence images of CMRA-labeled migrated CD8⁺ T cells (left panel). Scale bars: 50 μm. Quantification of migrated CD8⁺ T cells (right panel). *p < 0.05; **p < 0.01; ***p < 0.001. **(G-I)** Cytotoxicity and cytokine secretion of CD8⁺ T cells activated by antigen-presenting BMDCs.** (G)** Schematic overview of the experimental design. BMDCs, CD8⁺ T cells, and MC38 CRC cells were co-cultured at a 2:20:1 ratio. **(H)** Quantification of specific lysis of MC38 CRC cells by CD8⁺ T cells after 24-hour and 48-hour co-culture. **p < 0.01; ns, no significance. **(I)** Cytokine secretion levels of CD8⁺ T cells after 24-hour co-culture, as determined by ELISA. *p < 0.05; **p < 0.01; ns, no significance.

To assess the *in vitro* immunogenic effects of the two candidate proteins, HNRNPU and NCL plasmids were transfected into CHO-K1 cells (**Figure 6A**). GFP-positive CHO-K1 cells were selected through antibiotic treatment and further isolated by FACS sorting. Western blot analysis confirmed the successful overexpression of HNRNPU and NCL in CHO-K1 cells (**Figure 6B**). To investigate whether immune responses were elicited by HNRNPU and NCL, mouse bone marrow-derived dendritic cells and splenic CD8^+^ T cells were used for in vitro experiments. The antigens were applied to dendritic cells for 6 hours to evaluate their antigen uptake ability. Green fluorescence signals corresponding to HNRNPU and NCL were observed on the dendritic cells, indicating successful internalization of the antigens by dendritic cells (**Figure S5A**). Flow cytometry revealed that both candidate proteins significantly increased the expression of cell surface markers CD86, MHC I, and MHC II in mouse BMDC models (**Figure 6C**-**D**). Furthermore, transwell migration assays demonstrated that BMDCs pretreated with HNRNPU or NCL facilitated the migration of CD8^+^ T cells toward MC38 CRC cells (**Figure 6E**-**F**). Remarkably, both HNRNPU and NCL induced CD8^+^ T cell-mediated cytotoxicity against MC38 CRC cells (**Figure 6G**-**H**). Furthermore, the secretion levels of IFN-γ and TNF-α were significantly elevated (**Figure 6I**). These findings suggest that HNRNPU and NCL possess strong potential to elicit immune responses and exhibit anti-tumor effects in CRC.

### Evaluation of in vivo immune responses induced by candidate neoantigen proteins in colorectal cancer mouse models

**Figure 7:**
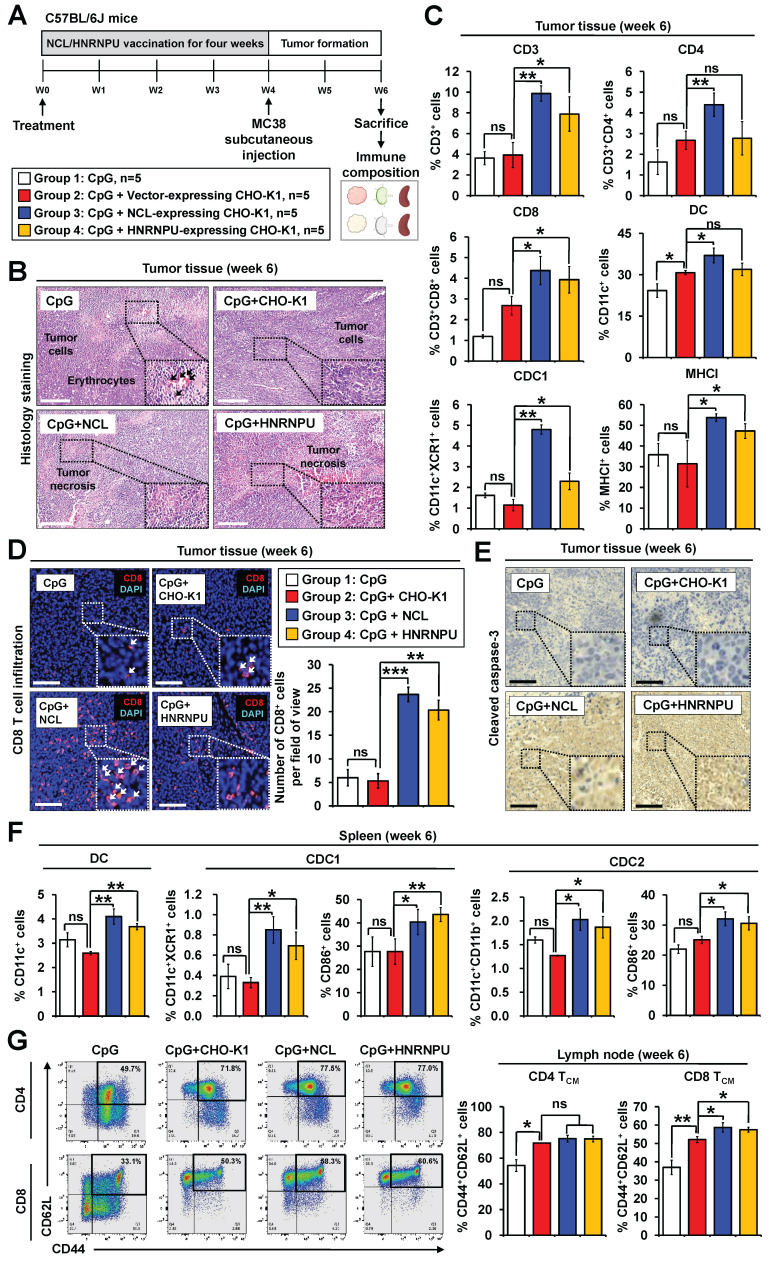
** Validation of immunogenicity of two candidate antigen proteins in colorectal cancer mouse models. (A)** Schematic overview of experimental design. PBS, 5 μM CpG, and 5 μM CpG plus CHO-K1 cell lysates (2 x 10^6^) were administered throughout the procedure.** (B)** Tumor sections were counterstained with H&E, and a high-magnification image of the area in the white box is shown. Scale bars: 250 μm.** (C)** Quantification of the population of T cells (CD45^+^CD3^+^), CD4^+^ T cells (CD45^+^CD3^+^CD4^+^), CD8^+^ T cells (CD45^+^CD3^+^CD8^+^), DCs (CD45^+^CD11c^+^), and cDC1 (CD45^+^CD11c^+^XCR1^+^) in tumor tissues. **(D)** Immunofluorescence analysis of CD8^+^ T cells (red fluorescence) in tumor sections. Nuclei were counterstained with DAPI (blue fluorescence). High-magnification images of boxed areas show CD8^+^ cells (white arrowheads). Scale bars: 200 μm. Quantification of CD8^+^ cells in tumor regions is shown. **p < 0.01; ***p < 0.001; ns, no significance. **(E)** Immunostaining of cleaved caspase-3 in tumor sections, with high-magnification images of boxed areas. Scale bars: 250 μm.** (F)** Vaccination with CHO-K1 lysates, HNRNPU-overexpressing lysates, or NCL-overexpressing lysates combined with CpG increased dendritic cell populations in mouse spleens. Populations included dendritic cells (CD45⁺CD11c⁺MHCII⁺), cDC1 (CD45⁺CD11c⁺MHCII⁺XCR1⁺), and cDC2 (CD45⁺CD11c⁺MHCII⁺CD11b⁺). Expression of costimulatory molecule CD86 on cDC1 and cDC2 was quantified, with representative histograms showing CD86 gating. *p < 0.05; **p < 0.01; ns, no significance.** (G)** Memory T cell populations in lymph nodes. Representative flow cytometry plots and quantification of CD4⁺ central memory T cells (CD4 TCM, CD4⁺CD44⁺CD62L⁺) and CD8⁺ central memory T cells (CD8 TCM, CD8⁺CD44⁺CD62L⁺) in mouse lymph nodes are presented. *p < 0.05; ns, no significance.

To investigate the* in vivo* immunogenic effects of HNRNPU and NCL, MC38 allograft mouse models were utilized (**Figure 7A**). At week 6, tumors, spleens, and lymph nodes were harvested and analyzed via flow cytometry to assess anti-tumor immune responses. Histological examination of tumor tissues demonstrated that treatment with either HNRNPU or NCL induced tumor necrosis in colorectal tumor regions (**Figure 7B**).

No apparent damage was observed in the histological examination of major organs, suggesting the safety of the vaccines (**Figure S5B**). NCL treatment significantly increased the proportions of T cells, CD4⁺ T cells, CD8⁺ T cells, dendritic cells, and conventional dendritic cell type 1 (cDC1) (**Figure 7C**). Similarly, HNRNPU treatment also led to a significant increase in T cells, CD4⁺ T cells, CD8⁺ T cells, and conventional dendritic cell type 1 (cDC1), but not in dendritic cells (**Figure 7C**). Notably, tumor-infiltrating CD8^+^ T cells were markedly elevated in the tumor regions of mice treated with HNRNPU or NCL (**Figure 7D**). Immunohistochemistry analysis further revealed that treatment with either HNRNPU or NCL induced cleaved caspase-3 expression in tumors (**Figure 7E**).

We investigated whether HNRNPU or NCL treatment could enhance immune cell populations in lymphoid organs, including the spleen and lymph nodes. Our findings revealed that the populations of dendritic cell subsets, cDC1 and conventional dendritic cells type 1 (cDC2), were significantly increased in the spleen (**Figure 7F**). These results suggest that HNRNPU and NCL treatment promotes the recruitment of dendritic cells to the spleen, a secondary lymphoid organ, thereby potentially facilitating T cell priming. To further explore long-term memory responses in vaccinated mice, we analyzed immune cell populations in tumor-draining lymph nodes. Our results demonstrated a significant increase in CD8^+^ central memory T cells (CD8^+^ TCM) but not in CD4^+^ central memory T cells (CD4^+^ TCM) in the treatment groups (**Figure 7G**). These findings indicate that NCL and HNRNPU treatment elicited robust long-term memory immune responses in CRC mouse models.

## Discussion

In this study, we employed an iPSC-based cancer vaccine in combination with an immune adjuvant to evaluate its prophylactic and therapeutic effects against CRC. The vaccine's capacity to prevent tumor initiation and treat established CRC was systematically assessed, along with its immune-stimulatory properties in CRC mouse models. In prophylactic studies, the iPSC-based vaccine effectively suppressed tumor initiation and elicited robust antitumor T cell and B cell responses. Importantly, it enhanced CD8^+^ T cell infiltration into CRC tumors and promoted T cell activation, leading to significant tumor prevention. In therapeutic studies, the iPSC-based vaccine inhibited CRC tumor growth by increasing CD8^+^ T cell infiltration and activating cleaved caspase-3, ultimately inducing tumor apoptosis. Notably, the vaccine exhibited therapeutic efficacy not only in primary CRC but also in metastatic CRC mouse models. Taken together, these findings underscore the effectiveness and immune-stimulatory potential of the iPSC-based cancer vaccine in combating CRC.

Although generating an autologous iPSC line for each patient may currently seem less feasible and a prophylactic cancer vaccine appears less relevant to clinical medicine, the iPSC-based cancer vaccine described in our study holds significant promise as a future immunotherapy in specific clinical scenarios. Firstly, establishing an autologous iPSC line for every patient is unnecessary, as hypoimmunogenic iPSCs can be generated by inactivating MHC I and MHC II genes, providing a universal iPSC transplantation source for potential clinical applications 37. Secondly, in a prophylactic context, the iPSC vaccine could be utilized to treat individuals at high risk of developing cancer, such as those with Lynch syndrome, IBD, Li-Fraumeni syndrome, chronic hepatitis B infection, hereditary chronic pancreatitis, or pathogenic germline mutations in BRCA1/2 genes 38-42. These populations have a significantly elevated lifetime risk of cancer, making them suitable candidates for prophylactic cancer vaccination. Thirdly, the iPSC vaccine also demonstrates therapeutic anti-tumor effects. In this study, we found that the iPSC vaccine demonstrated therapeutic efficacy in reducing tumor growth in both primary and metastatic CRC mouse models. This approach could be implemented at the time of cancer diagnosis and made available during surgical, chemo-, or radiotherapy treatments. Under these scenarios, the clinical development of the iPSC-based cancer vaccine described in our study is both warranted and feasible.

In this study, we did not isolate membrane proteins from iPSCs for antigen discovery, as we used whole-cell lysates of iPSCs and observed antitumor effects in mouse models, consistent with findings from our previous studies 15, 20. Theoretically, T cells are activated by recognizing peptides of antigenic proteins, rather than whole antigen proteins, bound to MHC molecules 43. According to the NetMHCpan-4.1 server prediction, only 7 to 11 peptides derived from HNRNPU and NCL proteins are predicted to be presented by MHC I. To confirm this, isolating MHC-bound peptides is worth further investigation. Evidence from human and murine systems demonstrates that cytotoxic T lymphocytes can recognize peptides derived from telomerase (TERT) and eliminate TERT-positive tumor cells across multiple histologies 44. Therefore, TERT has been considered a universal tumor antigen for cancer vaccines 45-47. Similar observations were made in this study. HNRNPU, a critical component of the nuclear matrix, is predominantly expressed in the cell nucleus 48. NCL is a multifunctional DNA and RNA-binding protein involved in the regulation of gene transcription and chromatin remodeling 49. NCL is localized in the nucleolus, nucleus, cytoplasm, and at the cell surface 50. We found that HNRNPU and NCL have strong potential to elicit immune responses and exhibit antitumor effects in CRC mouse models. In addition to mouse CRC models, analysis of the TCGA database revealed that transcriptional expressions of HNRNPU and NCL were significantly elevated in cancer tissues compared to normal tissues, not only in CRC but also in other types of human cancers, such as melanoma, breast, lung, and pancreatic cancers (**Figure S6A-C**). The NetMHCpan-4.1 server further identified a strong binding affinity between human MHC I and peptides derived from HNRNPU and NCL (**Figure S6D**). These findings suggest that HNRNPU and NCL may serve as universal tumor antigens for cancer vaccines.

Tumor-infiltrating T cells play a critical role in antitumor activity by eliminating malignant cells. However, prolonged activation inevitably leads to T cell exhaustion 51. Exhausted T cells are characterized by the expression of markers such as PD-1, CTLA-4, TIM-3, LAG-3, and TIGIT, along with diminished effector functions 52, 53. The enhanced antitumor efficacy of therapeutic cancer vaccines when combined with immune checkpoint inhibitors (ICIs) has been demonstrated in both preclinical and clinical studies. For instance, NEO-PV-01, a modified long-peptide vaccine formulated with poly ICLC, exhibited improved antitumor responses when combined with the anti-PD-1 antibody nivolumab in patients with advanced-stage melanoma, non-small cell lung cancer, or urothelial cancer 54, 55. In this study, elevated PD-L1 expression was observed in tumor sections from CRC mice treated with HNRNPU and NCL (**Figure S7**). Three anti-PD-L1 antibodies have been approved by the FDA: atezolizumab (Tecentriq^®^), durvalumab (Imfinzi^®^), and avelumab (Bavencio^®^). These antibodies block immune checkpoint interactions between T cells and cancer cells, thereby reactivating T cell functions 56. The combination of the iPSC-based vaccine and anti-PD-L1 antibodies has the potential to further enhance the therapeutic efficacy of the iPSC-based vaccine by augmenting antitumor immune responses in CRC, warranting further investigation.

Our previous data indicate that iPSCs alone did not significantly prevent the formation of various tumors in mice, including melanoma, breast, lung, and pancreatic cancers 15, 20. The addition of CpG was found to be essential for achieving tumor preventive effects in these tumor types. CpGs are unmethylated synthetic oligonucleotides (ODNs) that mimic microbial DNA, thereby activating Toll-like receptor 9 (TLR9) on antigen-presenting cells, such as macrophages, dendritic cells, and B cells 57. CpG has been extensively studied as an immune adjuvant in cancer vaccines, as it enhances the function of professional antigen-presenting cells and boosts the generation of cellular and humoral antigen-specific immune responses 58, 59. In this study, CpG was again utilized as the adjuvant, and the combination of CpG with iPSCs or candidate neoantigens (HNRNPU and NCL) demonstrated potent antitumor effects in CRC. Toll-like receptors (TLRs) represent the most common targets for cancer vaccine adjuvants 60. For instance, Pam3CSK4 targets TLR-1, TLR-2, and TLR-6, while poly(I:C) and lipopolysaccharide (LPS) stimulate TLR-3 and TLR-4, respectively 61. Further investigation is required to determine whether CpG is the optimal adjuvant for eliciting the most potent antitumor responses in combination with iPSCs.

**Figure 8:**
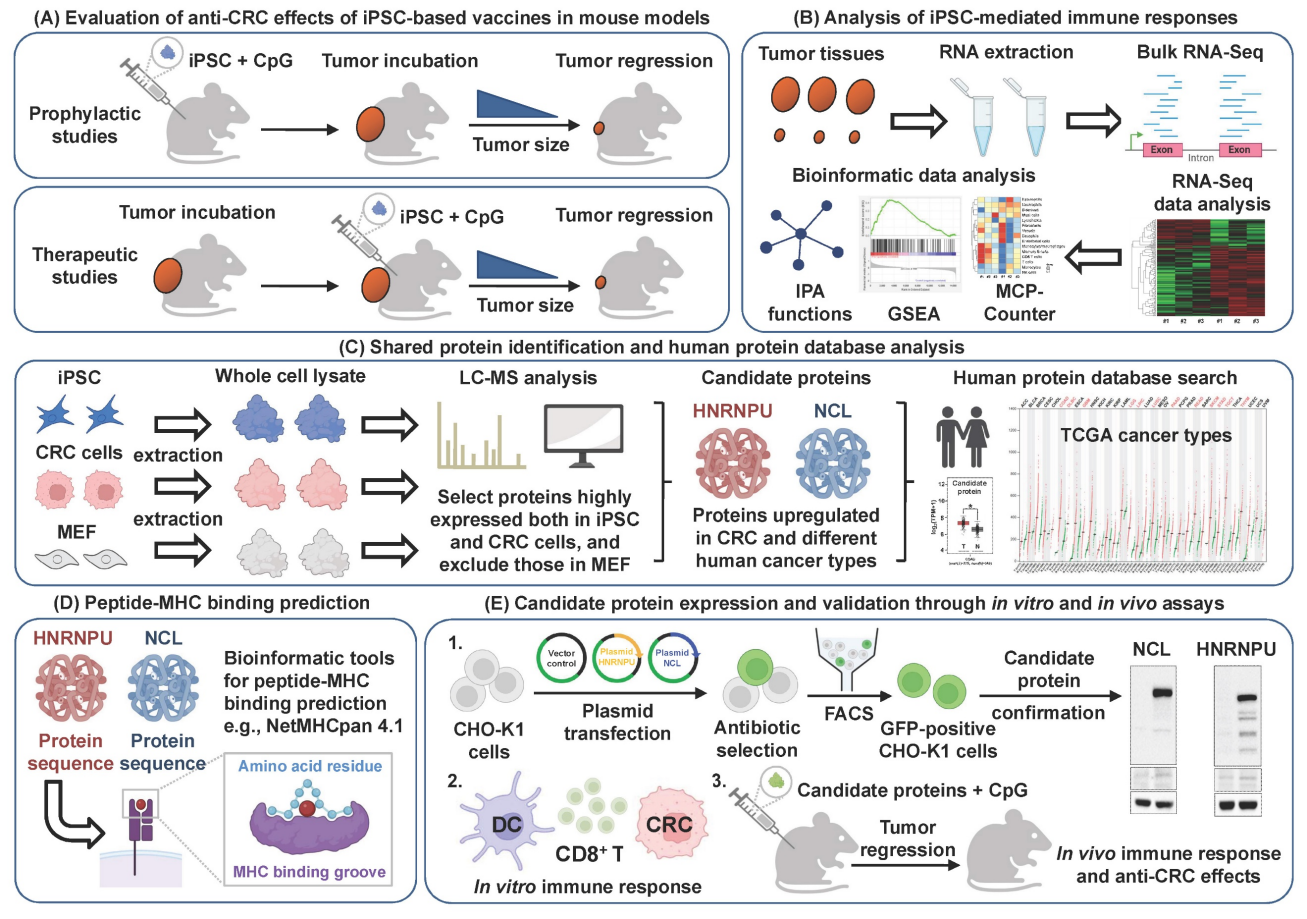
** Schematic illustration of candidate antigen protein prediction and validation to examine the anti-colorectal cancer effects of the iPSC-based vaccine. (A)** Evaluation of anti-CRC effects. Overview of the anti-CRC effects of iPSC-based vaccine in mouse models.** (B)** Immune response analysis. RNA-Seq and bioinformatics tools were used to analyze immune responses induced by the iPSC-based vaccine. **(C)** Protein identification and database analysis. Shared proteins between iPSCs and CRC cells were identified using LC-MS analysis, followed by further characterization using the human protein database.** (D)** Peptide-MHC binding prediction. In silico bioinformatics tools were employed to predict peptide-MHC binding affinity of candidate antigens.** (E)** Candidate protein expression and validation. Candidate proteins were overexpressed in CHO-K1 cells, and their immunogenic functions were investigated using both *in vitro* and *in vivo* models.

Taken together, our findings demonstrate the feasibility and efficacy of an iPSC-based vaccine for preventing and treating CRC through the activation of CD8^+^ T cells. We identified two iPSC-associated proteins, HNRNPU and NCL, as key mediators of the immune responses elicited by the iPSC-based vaccine. Vaccination with HNRNPU or NCL enhanced dendritic cell activation, induced antigen-specific CD8^+^ T cell cytotoxicity, and promoted the formation of central memory CD8^+^ T cells, ultimately leading to CRC tumor reduction. Moreover, these two iPSC-associated proteins are highly expressed not only in CRC but also in various types of human cancers, suggesting their potential as universal tumor antigens for cancer vaccine development (**Figure 8**).

## Supplementary Material

Supplementary figures and table, materials and methods.
