# Supplementary material for: Dual prophylactic and therapeutic potential of iPSC-based vaccines and neoantigen discovery in colorectal cancer
Source: Theranostics. 2025 Apr 28;15(12):5890–908. doi: 10.7150/thno.111400 (PMC12068288; doi:10.7150/thno.111400)
Supplement: Supplementary file 1 — Supplementary figures and table, materials and methods. [file thnov15p5890s1.pdf]

Supplementary Information

Dual prophylactic and therapeutic potential of iPSC-based vaccines and neoantigen discovery in colorectal cancer

Si-Han Jwo[1], Shang-Kok Ng[1], Chin-Tzu Li[1], Shao-Peng Chen[1], Li-Yu Chen[1], Pin-Jung Liu[2], Huai-Jie Wang[1], Jr-Shiuan Lin[3], Chun-Jung Ko[3], Cheng-Fan Lee[4], Chun-Hao Wang[5], Xiaoming Ouyang[6], Lin Wang[6], Tzu-Tang Wei[1, 7]*

Inventory of Supplementary Information

Supplementary Data

Figure S1, related to Figure 1

Figure S2, related to Figure 2

Figure S3, related to Figure 3

Figure S4

Figure S5, related to Figure 7

Figure S6

Figure S7, related to Figure 7

Table S1

Supplementary Materials and Methods

Figure S1

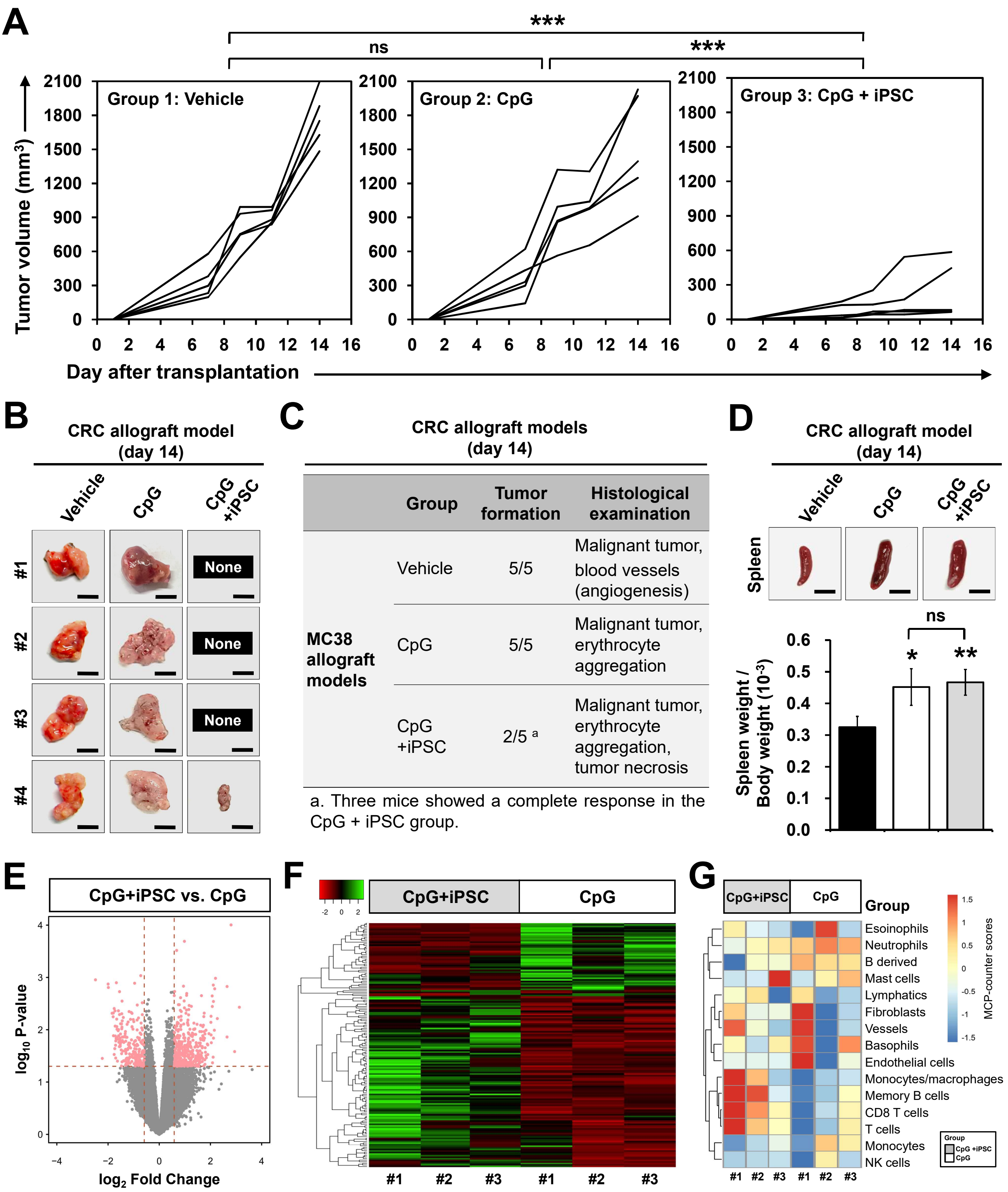
A
***
ns
***
Tumor volume (mm³)
Day after transplantation
Group 1: Vehicle
Group 2: CpG
Group 3: CpG + iPSC
0 300 600 900 1200 1500 1800 2100
0 2 4 6 8 10 12 14 16
B
CRC allograft model (day 14)
Vehicle
CpG
CpG +iPSC
#1
#2
#3
#4
None
C
CRC allograft models (day 14)
Group
Tumor formation
Histological examination
MC38 allograft models
Vehicle
5/5
Malignant tumor, blood vessels (angiogenesis)
CpG
5/5
Malignant tumor, erythrocyte aggregation
CpG +iPSC
2/5 a
Malignant tumor, erythrocyte aggregation, tumor necrosis
a. Three mice showed a complete response in the CpG + iPSC group.
D
CRC allograft model (day 14)
Vehicle
CpG
CpG +iPSC
Spleen
Spleen weight / Body weight (10⁻³)
ns
*
**
0.0 0.1 0.2 0.3 0.4 0.5 0.6
E
CpG+iPSC vs. CpG
log10 P-value
log2 Fold Change
F
CpG+iPSC
CpG
#1 #2 #3 #1 #2 #3
G
CpG+iPSC
CpG
Group
Esoinophils
Neutrophils
B derived
Mast cells
Lymphatics
Fibroblasts
Vessels
Basophils
Endothelial cells
Monocytes/macrophages
Memory B cells
CD8 T cells
T cells
Monocytes
NK cells
MCP-counter scores
#1 #2 #3 #1 #2 #3

Figure S1. Prophylactic effect of the iPSC-based vaccine in colorectal cancer allograft mouse models. Male C57BL/6 mice were vaccinated for 4 weeks with PBS (vehicle), CpG (5 μM), or CpG plus iPSCs (2×10^6). MC38 cells (2×10^6) were then subcutaneously implanted into the lower back, and tumor growth was measured every two days for two weeks. **(A)** Growth curves of individual MC38 tumors treated as indicated. Tumor volume was calculated using the formula: $V = 0.5 \times$ (length of the longest diameter) $\times$ (length of the shortest diameter)^2. Two-way ANOVA was used to define the p-value. *p < 0.05; **p < 0.01; ***p < 0.001; ns, no significance. **(B)** Gross images of allograft tumors at the end of the experiment. Scale bar: 1 cm. **(C)** Summary of MC38 allograft mouse model outcomes after iPSC vaccination. **(D)** Gross images of mouse spleens (upper panel) and excised spleen weights (lower panel) at the end of the experiment. Scale bar: 1 cm. *p < 0.05; **p < 0.01; ns, no significance. **(E-G)** Bulk RNA-Sequencing (RNA-Seq) analysis of tumor tissues from colorectal cancer allograft mouse models. **(E)** Volcano plot showing the adjusted p-value and fold-change for gene expression comparisons between CpG and CpG + iPSC groups. Genes with adjusted p-value < 0.05 and fold-change > 2 are highlighted. **(F)** Heatmap of the top 100 differentially expressed genes (DEGs) from CpG and CpG + iPSC groups. Gene expression levels were normalized by Transcript Per Million (TPM) and log-transformed. Hierarchical clustering highlights upregulated (red) and downregulated (green) genes induced by iPSC vaccination (n = 3 per group). **(G)** Murine Microenvironment Cell Population (MCP)-Counter analysis reveals differences in immune and stromal cell populations between CpG and CpG + iPSC groups. Heatmap displays fractions of cell populations, including B cells, cancer-associated fibroblasts, monocytes, macrophages, T cells, and activated $CD8^+$ T cells.

Figure S2

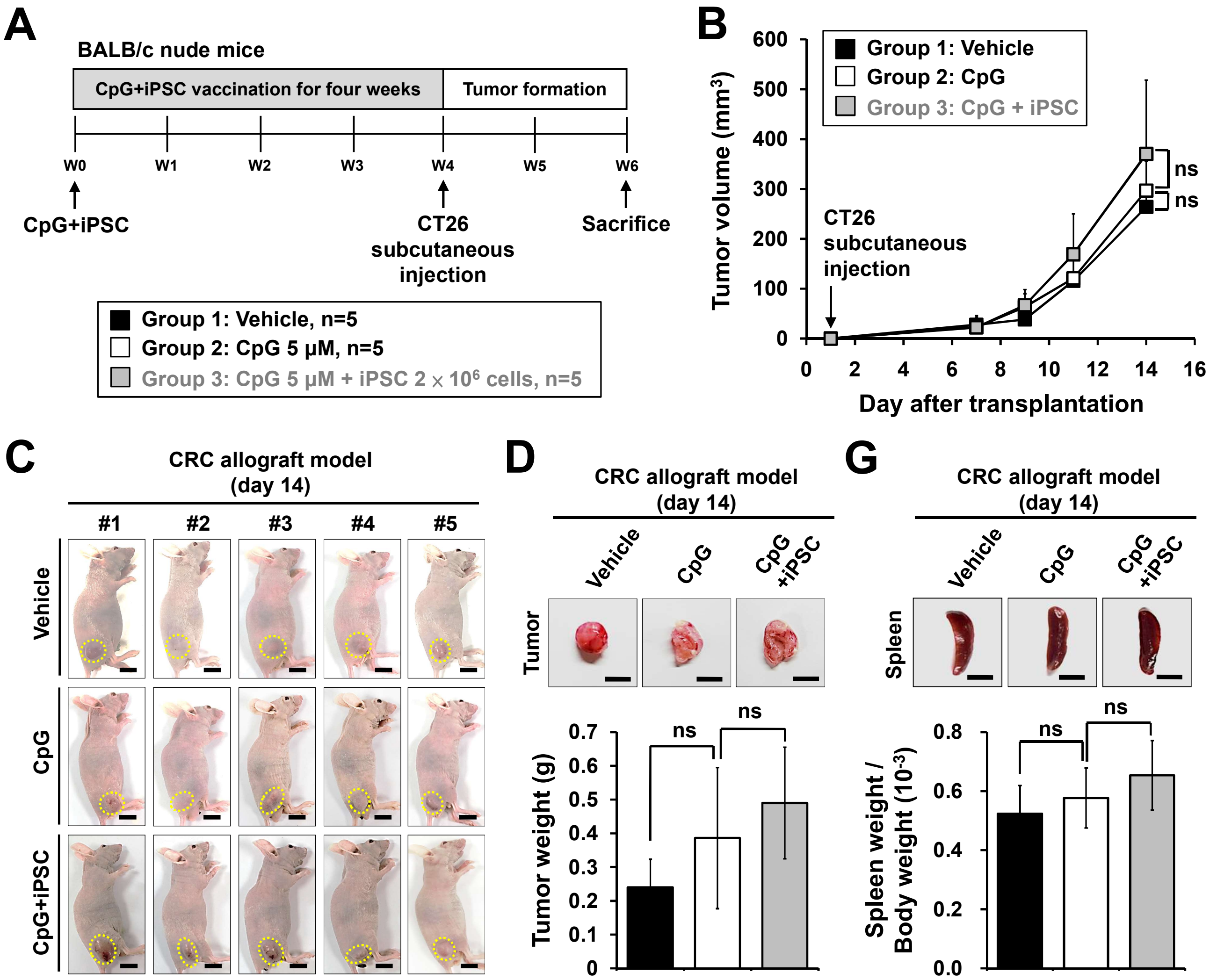

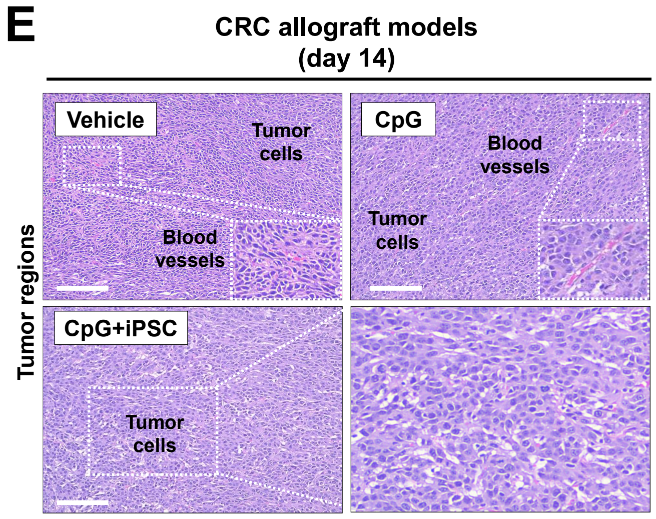

F

CRC allograft models
(day 14)

	Group	Tumor formation	Histological examination
**CT26 allograft models**	Vehicle	5/5	Malignant tumor, blood vessels (angiogenesis)
	CpG	5/5	Malignant tumor, blood vessels (angiogenesis)
	CpG+iPSC	5/5	Malignant tumor

Figure S2. Loss of iPSC-mediated prophylactic effects on colorectal cancer in immunodeficient mouse models. Male BALB/c nude mice were vaccinated for 4 weeks with vehicle (PBS), CpG (5 μM), or CpG plus iPSCs (2×10^6). Afterward, CT26 cells (2×10^6) were subcutaneously implanted into the lower back of BALB/c nude mice. The tumors were measured every two days for two weeks. **(A)** Schematic overview of the experimental design for the mouse models. **(B)** The tumor volume was calculated as follows: V = 0.5 × (length of the longest diameter) × (length of the shortest diameter)^2. Two-way ANOVA was used to define the p-value. ns, no significance. **(C)** Images of the tumor burden in each group. The yellow circle indicates the tumor region. Scale bar: 1 cm. **(D)** Gross images of allograft tumors are shown (upper panel). Scale bar: 1 cm. At the end of the experiment, the excised tumors were weighed (lower panel). ns, no significance. **(E)** Tumor sections from the mouse models were counterstained with H&E, and a high-magnification image of the area in the white box is shown. Scale bars: 250 μm. **(F)** Summary of the CT26 allograft mouse models after iPSC vaccination. **(G)** Gross images of mouse spleens are shown (upper panel). Scale bar: 1 cm. At the end of the experiment, the excised spleens were weighed (lower panel). ns, no significance.

Figure S3

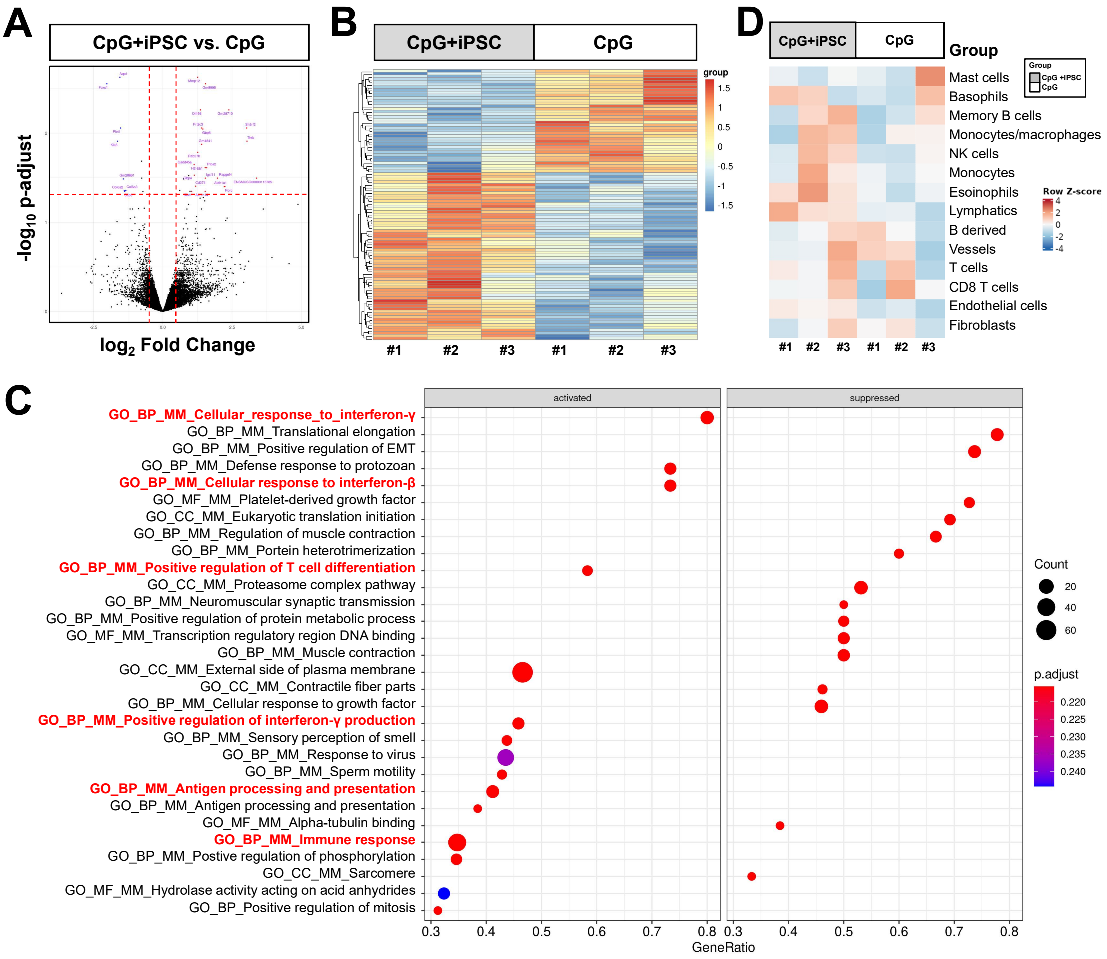

Figure S3. RNA sequencing analysis of tumor tissues from colorectal cancer allograft mouse models. (A) The volcano plot showed the adjusted p-value and fold-change for the comparisons of CpG versus CpG + iPSC groups, and the genes with adjusted p-value < 0.05 and fold change > 2 were highlighted. **(B)** Heatmap of differentially expressed genes (DEGs) from CpG and CpG + iPSC groups showing expression levels of the top 100 highly variant genes. Heatmap counts were normalized by Transcript Per Million (TPM) method and were displayed based on log normalize counts. Hierarchical clustering presents upregulated (red) and downregulated (green) genes induced by iPSC vaccination (n=3 per group). **(C)** Ingenuity Pathways Analysis (IPA) identifies immune response genes with significant molecular networks and over 2-fold expression. **(D)** The murine Microenvironment Cell Population counter (MCP-Counter) deconvolution revealed differences in the population of microenvironment cells such as B cells, cancer-associated fibroblast, monocytes, macrophages, T cells, and activated $CD8^{+}$ T cells in MC38 tumor tissues. Heatmap of mMCP-counter analysis displayed the fraction of immune and stromal cell population between CpG and CpG + iPSC groups.

Figure S4

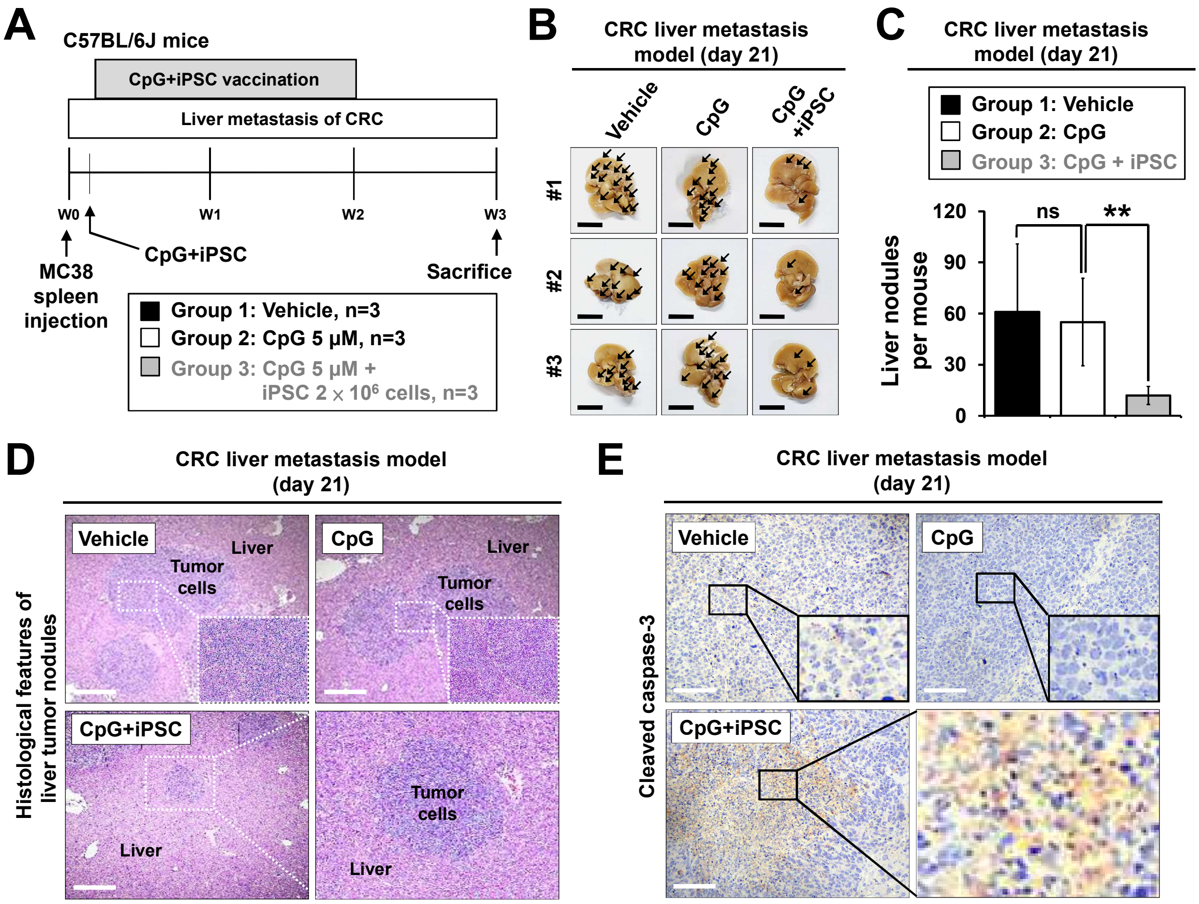

Figure S4. Therapeutic effects of the induced pluripotent stem cell-based vaccine in liver metastasis colorectal cancer mouse models. (A) Schematic overview of the experimental design. **(B)** Gross pictures of mouse liver are shown. The arrowheads indicate macroscopic tumor nodules (scale bar: 5 mm). **(C)** The number of liver nodules was graphed three weeks after iPSC-based vaccine treatment. **$p < 0.01$; ns, no significance. **(D)** Liver sections from the mouse models were counterstained with H&E, and a high-magnification image of the area in the white box is shown. Scale bars: 250 μm. **(E)** Liver tissues were immunostained with cleaved caaspase-3 antibodies, and a high-magnification image of the area in the black box is shown. Scale bar: 250 μm.

Figure S5

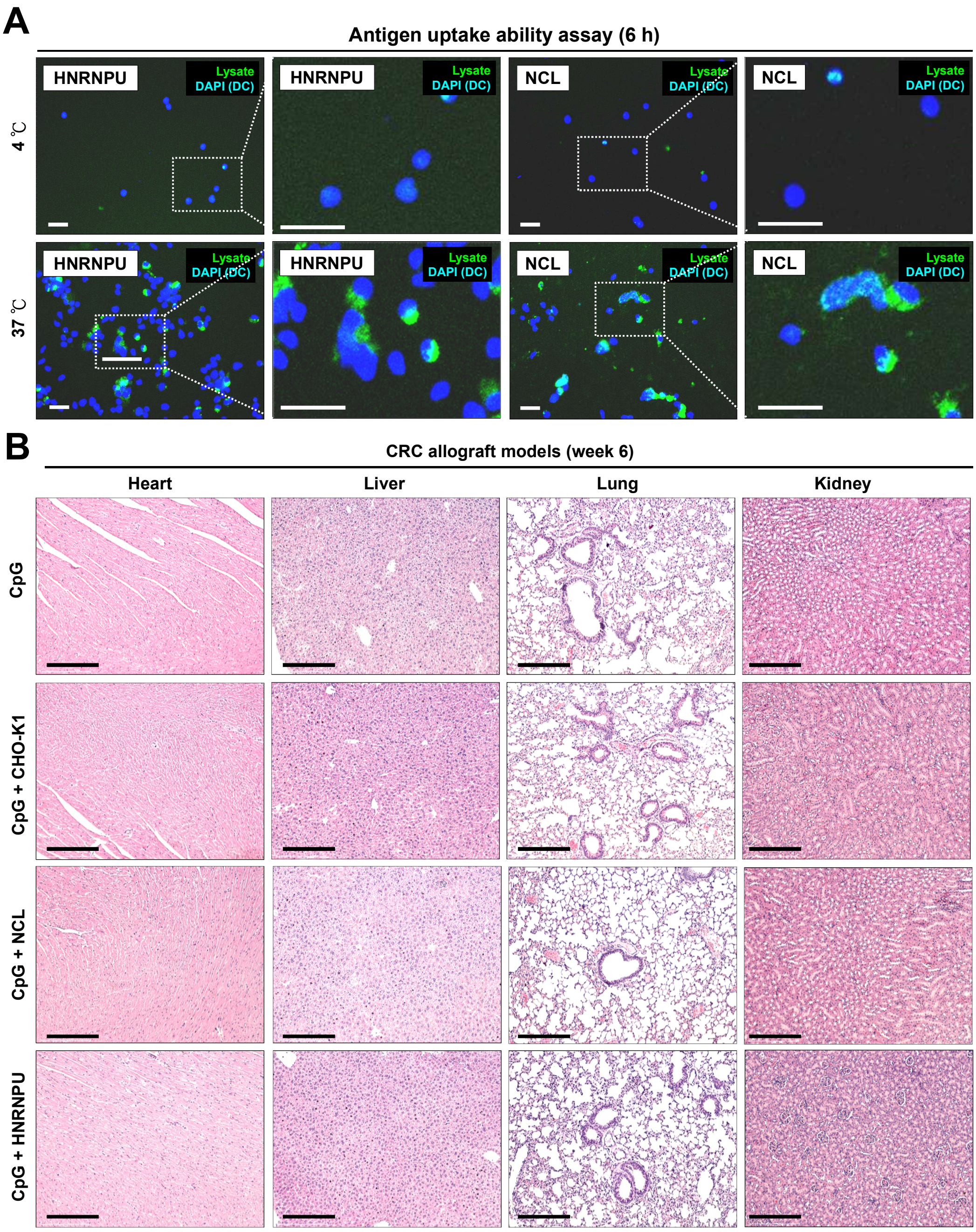

Figure S5. Effects of HNRNPU and NCL in colorectal cancer mouse models. (A) The antigen uptake ability of dendritic cells was assessed after 6 hours of antigen stimulation, with a 1:3 ratio of mouse bone marrow-derived dendritic cells (BMDCs) to cell lysates. Representative fluorescence images show cell lysates (green fluorescence) and nuclei, which were counterstained with DAPI (blue fluorescence). Scale bar: 75 μm. **(B)** Representative images of major organs from vaccine-treated CRC mice sacrificed at week 6. Three sections of each organ were counterstained with H&E, and one representative slide was presented. Scale bars, 250 mm.

Figure S6

A

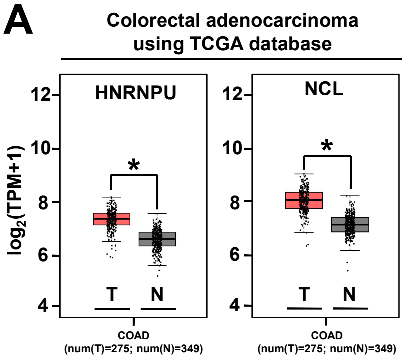

D

Prediction of MHC I binding epitopes on human iPSC-associated antigens using NetMHCpan-4.1 server

Allele	Peptide	Identity	Affinity (nM)	Binding level
HLA-B40:01	HEVRIGWSL	hHNRNPU	5.1	SB
HLA-B40:01	GEEEFSYGYSL	hHNRNPU	7.2	SB
HLA-A01:01	ESDEVELSY	hHNRNPU	7.69	SB
HLA-B07:02	RPAMEPGNGSL	hHNRNPU	7.9	SB
HLA-B58:01	KSSGPTSLF	hHNRNPU	9.19	SB
HLA-B40:01	REIEGRAIRL	hNCL	8.57	SB
HLA-B40:01	GEIDGNKVTL	hNCL	8.57	SB
HLA-B40:01	TEIDGRSISL	hNCL	12.11	SB

B

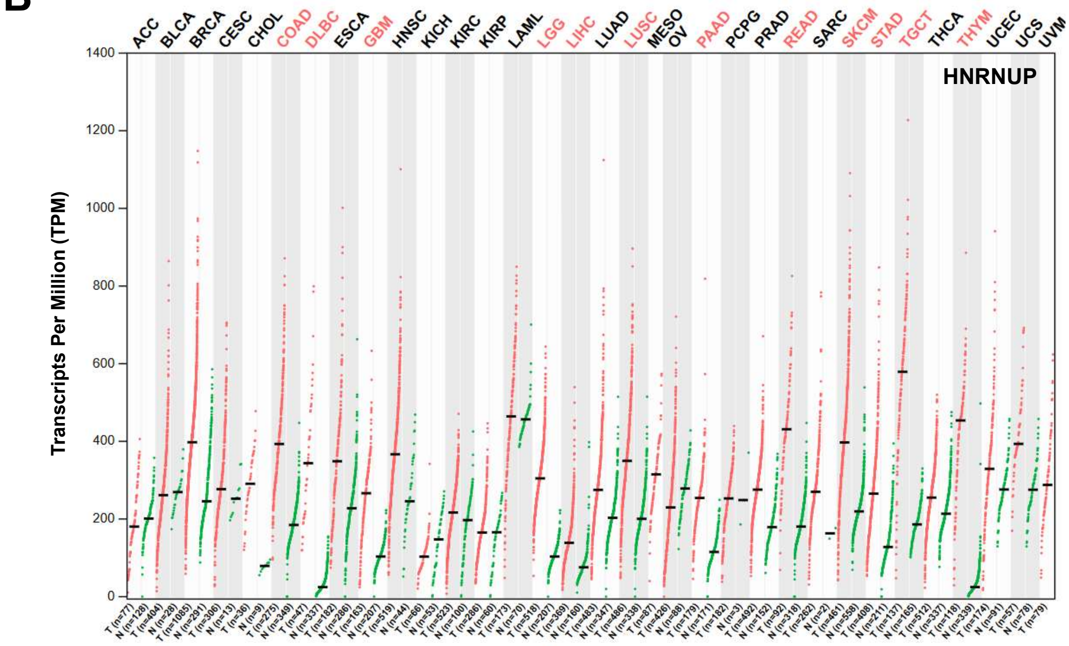

C

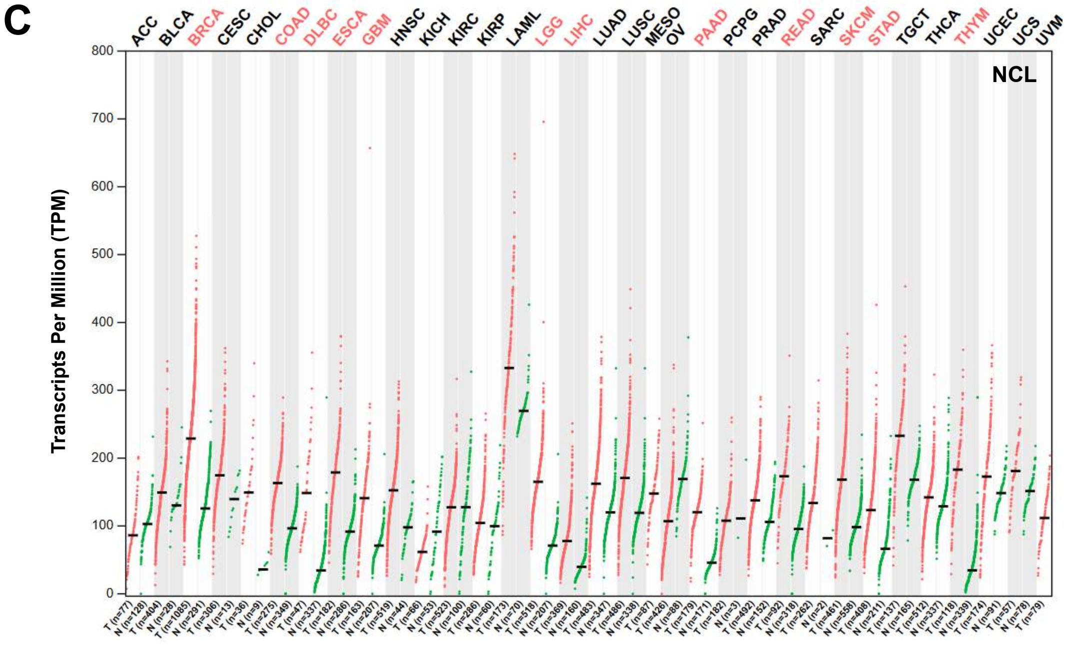

Figure S6. Expression levels of HNRNPU and NCL in human cancers. (A) (A) The expression levels of HNRNPU and NCL in colorectal adenocarcinoma (COAD) were analyzed using GEPIA (http://gepia.cancer-pku.cn/), comparing mRNA expression levels in normal and COAD tissues. $*p < 0.05$. **(B)** Expression levels of HNRNPU across 33 tumor types were determined using The Cancer Genome Atlas (TCGA) database (https://gdc.cancer.gov/). The red bars represent tumor samples, and the gray bars represent normal tissue samples. **(C)** Expression levels of NCL across 33 tumor types were also determined using the TCGA database. The red bars represent tumor samples, and the gray bars represent normal tissue samples. **(D)** Prediction of MHC I binding epitopes on potential antigens was performed using the NetMHCpan-4.1 server (https://services.healthtech.dtu.dk/services/NetMHCpan-4.1/). SB, strong binder.

Figure S7

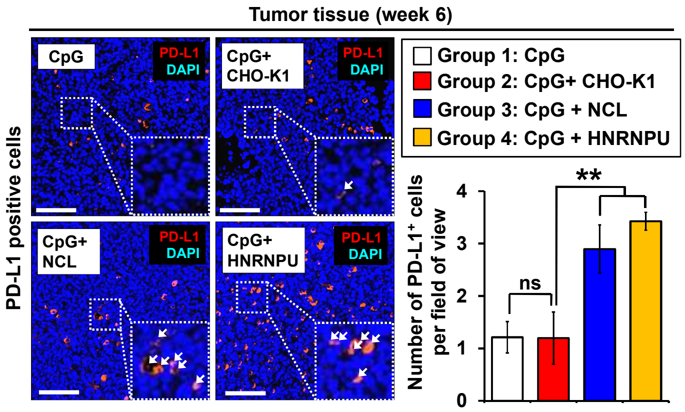

Figure S7. Expressions of PD-L1 in colorectal mouse models. The expression of PD-L1$^+$ T cells (red fluorescence) in tumor sections was assessed by immunofluorescence, with nuclei counterstained using DAPI (blue fluorescence). High-magnification images of the areas marked by white boxes are shown. The white arrowhead indicates PD-L1$^+$ cells (left panel). Scale bar: 200 μm. The number of PD-L1$^+$ cells in tumor regions was quantified (right panel). **$p < 0.01$; ns, no significance.

Table S1. List of antibodies used in this study.

Antibodies	Supplier name	Catalog number
Anti-β-actin Ab	GeneTex	GTX109639
Anti-Caspase 3 Ab	Abclonal	A11953
Anti-Cleaved caspase 3 Ab	Cell Signaling	9661S
Anti-CD4 Ab	Abcam	ab183685
Anti-CD45 (BV421) Ab	BD Biosciences	563890
Anti-CD45 (PerCP) Ab	BD Biosciences	561047
Anti-CD19 (APC-R700) Ab	BD Biosciences	565473
Anti-CD3 (FITC) Ab	BD Biosciences	561827
Anti-CD3 (BUV395) Ab	BD Biosciences	563565
Anti-CD4 (PE) Ab	BD Biosciences	561829
Anti-CD8 (APC) Ab	BD Biosciences	561093
Anti-CD62L (BUV395) Ab	BD Biosciences	740218
Anti-CD44 (BUV737) Ab	BD Biosciences	612799
Anti-CD25 (BV605) Ab	BD Biosciences	563061
Anti-CD11c (PE-Cy7) Ab	BD Biosciences	561356
Anti-CD86 (BV421) Ab	BD Biosciences	564198
Anti-CD8 Ab	GeneTex	GTX74778
Anti-CD8 Ab	Abcam	ab209775
Anti-GAPDH	Cell Signaling	2118S
Anti-GFP Ab	Santa Cruz	sc-9996
Anti-Granzyme B Ab	Abclonal	A2557
Anti-HNRNPU Ab	Abclonal	A4257
Anti-IFN-γ Ab	Abclonal	A12450

Anti-IL-6 Ab	Abclonal	A0286
Anti-IL-10 Ab	Abclonal	A20723
Anti-MHCI (BUV395) Ab	BD Biosciences	749705
Anti-MHCI (BV510) Ab	BD Biosciences	749710
Anti-MHCII (BV650) Ab	BD Biosciences	563415
Anti-NCL Ab	Abclonal	A5904
Anti-TNFα- Ab	Abclonal	A0277
Anti-XCR1 (BV510) Ab	Biolegend	148218

SUPPLEMENTARY MATERIALS AND METHODS

Reagents

Dimethyl sulfoxide (DMSO, D2650), azoxymethane (AOM, A5486), and Formaldehyde solution 4% buffer (1.00496) were purchased from Sigma-Aldrich (Burlington, MA, USA). Dextran sulfate sodium (DSS) was purchased from MP Biomedicals (9011-18-1, Solon, OH, USA). CpG ODN 1826 VacciGrade was purchased by InvivoGen (vac-1826-1, San Diego, CA, USA).

Cell culture

C57BL/6J mouse iPSCs were kindly provided by Dr. Joseph C. Wu (Stanford Cardiovascular Institute, USA) and Dr. Yaa-Jyuhn James Meir (Chang Gung University, Taiwan). Mouse iPSCs were cultured in 0.2% gelatin-coated plates and grown in KnockOut DMEM (Thermo Fisher Scientific, 10829018, Waltham, MA, USA) with 5 % KnockOut Serum Replacement (Thermo Fisher Scientific, 10828028), 2mM GlutaMAX Supplement (Thermo Fisher Scientific, 35050061), 1x MEM Non-Essential Amino Acids (Thermo Fisher Scientific, 11140050), 100 μM 2-Mercaptoethanol (Thermo Fisher Scientific, 21985023), 0.5 μM PD0325901 (ERK inhibitor, Selleck Chemicals, S1036, Houston, TX, USA), 3 μM CHIR-99021 (GSK-3β Inhibitor, Selleck Chemicals, S1263) and 10 ng/ml mouse recombinant LIF protein (Sigma-Aldrich, 665781), which add freshly every day. With every passage, AccutaseTM (Sigma-Aldrich, A6964) was used as cell dissociation reagent, and 10 μM Y-27632 (Rho-associated protein kinase inhibitor, Selleck Chemicals, S1049) was added on the first day of passage. iPSCs were incubated at in a 37 °C incubator containing 20% O_2 and 5% CO_2. The medium should be refreshed every day. The colorectal cancer cell line MC38 was provided by Dr. Zi-Ming Pan (National Taiwan

University, Taiwan). MC38 cells were cultured in DMEM with 10% fetal bovine serum (FBS; Thermo Fisher Scientific, A5256701) and 1% penicillin-streptomycin (Thermo Fisher Scientific, 15140122).

RNA extraction from tumor tissues

Mouse tumor tissues were frozen in liquid nitrogen and homogenized by grinding in TRIzol RNA Isolation Reagents (Thermo Fisher Scientific, 15596026). The samples incubated in QIAzol for 5 minutes and were centrifuged at 11000 rpm for 10 minutes at 4 °C and repeated twice to remove lysed tissues. 200 μL chloroform was added, mixed vigorously and incubated for 3 minutes before centrifuging at 11000 rpm for 15 minutes. RNA was dissolved in the upper aqueous and was then precipitated with 500 μL isopropanol for 10 minutes. After centrifuged at 11000 rpm for 10 minutes, the pellet was washed with ethanol and spun down at 8000 rpm for 5 minutes and repeated twice. The pellet was air dried and dissolved in 10-20 μL nuclease-free water. RNA concentration was measured using Nanodrop spectrophotometer. cDNA was synthesized with Maxima First Strand cDNA Synthesis Kit (Thermo Fisher Scientific, K1641) according to the manufacturer's protocol.

Flow cytometry

Cells were treated with TruStain Fc anti-mouse CD16/32 Antibody (Fc blocker, Biolegend, 101320, San Diego, CA, USA) and dead cells were excluded using BD Horizon Fixable Viability Stain 620 (BD Biosciences, FVS620, Becton, NJ, USA). Cells were stained with surface marker antibodies for 20 minutes on ice and fixed with 2% paraformaldehyde (PFA) for 30 minutes on ice. BD LSRFortessa Cell Analyzer (BD Biosciences) and Cytek Aurora (Cytek Biosciences, Fremont, CA, USA) were used for flow cytometry and the data were analyzed using FlowJo

software (BD Biosciences, v10.8.1 version). For cell sorting, cells were sorted by BD FACSAri III Cell Sorter (BD Biosciences).

Enzyme-linked immunosorbent assay (ELISA)

The productions of TNF-α and IFN-γ in the supernatants of cultured $CD8^+$ T cells were detected using ELISA MAX Deluxe Set Mouse TNF-α (Biolegend, 430904) and ELISA MAX Deluxe Set Mouse IFN-γ kit (Biolegend, 430804). The assays were performed according to the manufacturer's instructions.

Animal model of AOM/DSS-induced CRC

Seven-week-old male C57BL/6 mice were obtained from the National Laboratory Animal Center (Taiwan). CRC was induced by intraperitoneal injection of AOM (12.5 mg/kg) in conjunction with DSS stimulation. Mice were maintained on a regular diet and drinking water for 7 days and were then subjected to 3 cycles of DSS treatment, with each cycle consisting of the administration of 3.5% DSS for 5 days followed by a 14-day recovery period with regular water. Colon tissues were collected and fixed with formalin. Mice were treated in accordance with protocols approved by the Institutional Animal Care and Use Committee of the College of Medicine, National Taiwan University.

Animal model for experimental CRC liver metastasis

Seven-week-old female NOD/SCID micewere anesthetized by a continuous flow of 2%-3% isoflurane. To generate the mouse models with liver metastases derived from human colorectal cancer cells, MC38-Luciferase-expressing cells (1×10^6) were suspended in 100 μL PBS and injected into the spleen of mice. After a one-day recovery, the mice were randomized into vehicle or treatment groups. Mice were then

given endotoxin-free luciferase substrate and photographed by IVIS imaging system (Xenogen) once a week. Mice were sacrificed, and tumors fromtheir spleens and liverswere collected, weighed, and fixed by formalin.

Immunohistochemical (IHC) staining

IHC staining was performed by TnAlink Polymer Detection System (BIOTnA Biotech, TAHC04A, Kaohsiung City, Taiwan). Mouse tissues were fixed in 4% formaldehyde. The paraffin-embedded tissue slides were heated at 37 °C incubator 1 hour and dewaxed in Sub-X xylene substitute (Leica Biosystems, 10015-094, Nussloch, Germany) 1 hour. The tissues were rehydrated in 100%, 95%, 90%, 80%, 70% alcohols and deionized water for 3 minutes each. The slides were then antigen retrieved with citric acid buffer at 95-100 °C for 20 minutes, followed by endogenous peroxidase block for 10 minutes and protein block for 90 minutes. The tissues incubated with primary antibody at 4 °C overnight. The tissues were rinsed with PBS three times and incubated with TAlink Polymer for 1 hour. After three times PBS washed, the sections were covered with DAB solution and counterstained with hematoxylin. The slides were rinsed in running water for 15 minutes and were dehydrated by gradually increasing the concentration of alcohol and xylene, and finally coverslipped with mounting medium. The specimens were imaged via EVOS M7000 Imaging System (Thermo Fisher Scientific).
